# The Ribosomal Protein L5 Functions During *Xenopus* Anterior Development Through Apoptotic Pathways

**DOI:** 10.3389/fcell.2022.777121

**Published:** 2022-02-22

**Authors:** Corinna Schreiner, Bianka Kernl, Petra Dietmann, Ricarda J. Riegger, Michael Kühl, Susanne J. Kühl

**Affiliations:** ^1^ Institute of Biochemistry and Molecular Biology, Ulm University, Ulm, Germany; ^2^ International Graduate School in Molecular Medicine Ulm, Ulm, Germany

**Keywords:** RPL5, *Xenopus laevis*, ribosomal biogenesis, ribosomopathy, c-myc, tp53

## Abstract

Ribosomal biogenesis is a fundamental process necessary for cell growth and division. Ribosomal protein L5 (Rpl5) is part of the large ribosomal subunit. Mutations in this protein have been associated with the congenital disease Diamond Blackfan anemia (DBA), a so called ribosomopathy. Despite of the ubiquitous need of ribosomes, clinical manifestations of DBA include tissue-specific symptoms, e.g., craniofacial malformations, eye abnormalities, skin pigmentation failure, cardiac defects or liver cirrhosis. Here, we made use of the vertebrate model organism *Xenopus laevis* and showed a specific expression of *rpl5* in the developing anterior tissue correlating with tissues affected in ribosomopathies. Upon Rpl5 knockdown using an antisense-based morpholino oligonucleotide approach, we showed different phenotypes affecting anterior tissue, i.e., defective cranial cartilage, malformed eyes, and microcephaly. Hence, the observed phenotypes in *Xenopus laevis* resemble the clinical manifestations of DBA. Analyses of the underlying molecular basis revealed that the expression of several marker genes of neural crest, eye, and brain are decreased during induction and differentiation of the respective tissue. Furthermore, Rpl5 knockdown led to decreased cell proliferation and increased cell apoptosis during early embryogenesis. Investigating the molecular mechanisms underlying Rpl5 function revealed a more than additive effect between either loss of function of Rpl5 and loss of function of c-Myc or loss of function of Rpl5 and gain of function of Tp53, suggesting a common signaling pathway of these proteins. The co-injection of the apoptosis blocking molecule Bcl2 resulted in a partial rescue of the eye phenotype, supporting the hypothesis that apoptosis is one main reason for the phenotypes occurring upon Rpl5 knockdown. With this study, we are able to shed more light on the still poorly understood molecular background of ribosomopathies.

## Introduction

Cell growth and division are fundamental for the development of any multicellular organism. These processes are highly regulated and hence, during embryogenesis, each dividing cell requires an adequate number of ribosomes to cope with the demand for translation. This demand is ensured by ribosome biogenesis, which mainly takes place in the nucleolus and nucleus. It requires around 200 factors and the three RNA polymerases, Pol I, Pol II, and Pol III, for pre-rRNA transcription, pre-rRNA processing, and ribosome assembly (Melnikov et al., 2012; Baßler and Hurt, 2019; Pecoraro et al., 2021).

Defects in ribosome biogenesis can lead to congenital diseases called ribosomopathies (Narla and Ebert, 2010; Farley-Barnes et al., 2019; Kang et al., 2021). Ribosomopathies such as the Diamond Blackfan anemia (DBA) or the Shwachman Diamond syndrome include various clinical manifestations. Regardless of the ubiquitous need of ribosomes in every cell of every organism, symptoms are often tissue-specific and include defects in craniofacial morphology, cardiac defects, skin pigmentation failure, bone marrow failure, and neurological impairments (Shwachman et al., 1964; Narla and Ebert, 2010; Brooks et al., 2014; Myers et al., 2014; Ross and Zarbalis, 2014; Jenkinson et al., 2016; Kostjukovits et al., 2017; Vlachos et al., 2018; Aspesi and Ellis, 2019; Farley-Barnes et al., 2019). Additionally, almost all ribosomopathies have a predisposition to develop tumors and eventually cancer (De Keersmaecker et al., 2015). Several genes have been identified whose mutations lead to impaired pre-rRNA transcription, pre-rRNA processing, or ribosome assembly (Valdez et al., 2004; Weaver et al., 2015; Jenkinson et al., 2016; Kostjukovits et al., 2017; Warren, 2018).

The ribosomal protein L5 (Rpl5) is one of those genes identified. Together with the ribosomal protein L11 (Rpl11) and the 5S rRNA, Rpl5 forms the 5S-ribonucleoprotein (RNP) complex, which is part of the 60S ribosomal subunit (Zhang and Lu, 2009; Leidig et al., 2014). Mutations in *rpl5* and the loss of Rpl5 function give rise to DBA in human (Gazda et al., 2008; Cmejla et al., 2009; Quarello et al., 2010).

As a consequence of disturbed ribosomal biogenesis, nucleolar stress and the subsequent increased number of free ribosomal proteins, the molecule MYC proto-oncogene (c-Myc) is affected. c-Myc enhances the transcriptional performance of all three RNA polymerases I-III crucial for ribosomal biogenesis and hence intensively contributes to this biological process (Gomez-Roman et al., 2003; Arabi et al., 2005; van Riggelen et al., 2010). It was shown in human cells, that upon cellular stress induced by defective ribosomal biogenesis, free ribosomal proteins Rpl5 and Rpl11 accumulate and can bind to *c-myc* RNA and induce *c-myc* RNA degradation by transporting it to the RNA-induced silencing complex (RISC). Consequently, *c-myc* RNA levels are reduced upon *rpl5* overexpression and increased upon Rpl5 knockdown (Liao et al., 2014). As a regulator of the neural crest, c-Myc reduction has been shown to lead to malformed cranial cartilages in *Xenopus* embryos (Bellmeyer et al., 2003).

A second molecule regulated by free ribosomal proteins is Tumor protein p53 (Tp53). In several human cell lines, free ribosomal proteins induce an activation of Tp53. This occurs by binding of 5S-RNP or free Rpl5 or/Rpl11 to and inactivation of the key regulator mouse double minute 2 homolog (MDM2). As a result, Tp53, that is not degraded by MDM2, accumulates and activates the pro-apoptotic pathway thereby contributing to the pathology of ribosomopathies (Dai and Lu, 2004; Zhang and Lu, 2009; Fumagalli et al., 2012; Sulima et al., 2019). Several studies in mice, zebrafish, and frogs have shown that craniofacial phenotypes, typical phenotypes for a ribosomopathy, can be rescued upon reducing Tp53 levels (Jones et al., 2008; Zhao et al., 2014; Griffin et al., 2015; Calo et al., 2018).

During early development, *Xenopus laevis* embryos contain a maternal store of mRNAs, proteins, and ribosomes (Wallace, 1960; Brown, 1964). This allows the embryo to be independent of *de novo* ribosomal biogenesis until stage 26 which has been shown by anucleolated mutants, that are not able to synthesize ribosomes, but can survive until swimming tadpole stage (Brown, 1964; Pierandrei-Amaldi and Amaldi, 1994). Although, *Xenopus laevis* seems to be independent of ribosomal biogenesis, RNA for ribosomal proteins, e.g., *rpl5* RNA, is detected throughout the early embryonic development (Pierandrei-Amaldi and Amaldi, 1994; Session et al., 2016; Briggs et al., 2018). Hence, the embryo contains *rpl5* RNA at developmental stages, during which it does not require *de novo* ribosomal biogenesis. This raises the question of whether Rpl5 has a function starting earlier than the start of *de novo* ribosomal biogenesis.

The aim of the following study was to investigate a potential function of Rpl5 during early anterior development of *Xenopus laevis* and to analyze whether we can recapitulate any phenotypes of ribosomopathies in this model organism. Therefore, expression analysis of *rpl5* as well as tissue-specific knockdown approaches *via* antisense-based morpholino oligonucleotides (MO) were performed. The molecular basis underlying the Rpl5 knockdown-induced phenotype was investigated by analyzing tissue-specific marker genes of the eye, the brain, and the neural crest, and proliferation as well as apoptosis. Additionally, the molecular mechanism was investigated by exploring the effects of Rpl5 knockdown on the two molecules Tp53 and c-Myc.

## Materials and Methods

### Xenopus laevis

*Xenopus leavis* embryos were generated, cultured and staged according to standard protocols (Nieuwkoop and Faber, 1954; Sive et al., 2000). All procedures were performed according to the German animal use and care law and approved by the German state administration Baden-Württemberg (Regierungspräsidium Tübingen). Embryos were cultivated in 0.1 × Modified Barth´s saline with HEPES buffer (MBSH) and fixed with MEMFA(T) [0.1 M MOPS (pH 7.4), 2 mM EGTA, 1 mM MgSO_4_, 4% formaldehyde, (0.1% Tween20)].

### Synteny Analysis and Protein Alignment of *Ribosomal Protein L5*

Synteny analysis and protein alignment of Rpl5 were performed using NCBI Gene Bank for *Homo sapiens* (NP_000960; Gene ID: 6125); *Mus musculus* (NP_058676; Gene ID: 100503670); *Gallus gallus* (NP_989912; Gene ID: 395269); *Danio rerio* (NP_956050; Gene ID: 326961); and the Xenbase platform (xenbase.org) for *Xenopus laevis* [NP_001079377; Gene ID: XB-GENE-6251827 (rpl5.S); NP_001079437; Gene ID: XB-GENE-983917 (rpl5.L)] and for *Xenopus tropicalis* (NP_988881; Gene ID: XB-GENE-983912). For protein alignments, the online tool NCBI protein blast was used.

### Morpholino Oligonucleotides, Cloning, and Microinjections

An Rpl5 morpholino oligonucleotide (MO) with the sequence 5′-CAT TTT GCT CTA TTT TGT CCC GTC G -3′ was designed, targeting the 5′UTR of *Xenopus laevis rpl5*. MOs for c-Myc and Tp53 were used as previously described (Bellmeyer et al., 2003; Cordenonsi et al., 2003). The gene-specific MOs and a standard Control MO were obtained from Gene Tools (Philomath, OR, United States). The MOs were diluted in diethyl pyro carbonate (DEPC) treated water.

To proof the binding specificity of Rpl5 MO, the MO binding sites were cloned in frame with and in front of the *GFP* (*green fluorescent protein*) gene as previously described (Gessert et al., 2007) using the following sequences:

Rpl5_MO_bs_GFP_l: 5′-GAT CCC GAC GGG ACA AAA TAG AGC AAA ATG GGG-3′,

Rpl5_MO_bs_GFP_r: 5′-AAT TCC CCA TTT TGC TCT ATT TTG TCC CGT CGG-3′,

Δ5′Rpl5_MO_bs_GFP_l: 5′-GAT CCA CTT GTT CTT TTT GCA GGA TCC ATG GGG-3′,

Δ5′Rpl5_MO_bs_GFP_r: 5′-AAT TCC CCA TGG ATC CTG CAA AAA GAA CAA GTG-3′,

1 ng of the respective MO-GFP RNA fusion construct was injected bilaterally together with 10 ng of Rpl5 MO, or Control MO into embryos at the two-cell stage and GFP expression was checked with an Olympus MVX10 fluorescence microscope.

If not indicated otherwise, 15–20 ng Rpl5 MO, 5 ng c-Myc MO, 2.5–5 ng Tp53 MO, or 15–20 ng Control MO were injected into one animal-dorsal blastomere of eight-cell embryos targeting anterior neural tissue (Moody and Kline, 1990). 0.5 ng *GFP* RNA was co-injected and served as injection control. The un-injected side served as internal control. To adjust the amount of RNA or MO per injection, *GFP* RNA and Control MO were used, respectively.

For rescue attempts, an *rpl5* construct was cloned with the following primers: rpl5_Bam_l: 5′-GGA TCC ATG GGG TTC GTA AAG GTC GTC AAG-3′ and rpl5_Bam_r: 5′-GGA TCC TTA GCT GTC TGC CTT CTC CTG AG-3′. This rescue construct is not targeted by the Rpl5 MO due to an altered sequence in the 5′UTR region. Rescue experiments were performed by co-injecting Rpl5 MO with 0.5 ng *rpl5* RNA. *c-myc* RNA, *tp53* RNA, and *human B cell lymphoma 2 (hBCL2)* RNA for injection were used as previously described (Bugner et al., 2011; Hampp et al., 2016).

Experiments which tested effects of low doses were carried out by injecting 5 ng Rpl5 MO and 0.5 ng *tp53* RNA unilaterally alone or in combination; furthermore, by injecting 5 ng Rpl5 MO and 5 ng c-Myc MO unilaterally alone or in combination.

### Whole Mount *In Situ* Hybridization

WMISHs were performed according to established protocols (Hemmati-Brivanlou et al., 1990; Lufkin, 2007). Digoxygenin-labeled antisense RNA probes were generated against different mRNAs by using T7, T3, or SP6 RNA polymerase (Roche). We cloned the open reading frame of *Xenopus laevis rpl5* into the pSC-B vector (Stratagene) with the cloning primers rpl5_l: 5′-CGT TTG GGC TGT GAC TAT CCG GTC-3′ and rpl5_r: 5′-TTA GCT GTC TGC CTT CTC CTG AGC-3′. *In vitro* transcription with T3 RNA polymerases (Roche) resulted in digoxygenin-labelled antisense RNA probes. Furthermore, we cloned the open reading frame of *Xenopus laevis tp53* into the pCS2+ vector (Rupp and Weintraub) with the cloning primers tp53_l: 5′-GGG ATC CAT GCT GAG A-3′ and tp53_r: 5′-AAG GCC TCA TGG CTG T-3′. *In vitro* transcription with T7 RNA polymerase (Roche) resulted in digoxygenin-labelled antisense RNA probes. We used the following RNA anti-sense probes as described previously: *hba3 (hemoglobin alpha 3 subunit)* (cDNA clone MGC:64476 IMAGE:6881400), *actc1 (actin, alpha, cardiac muscle 1)* (cDNA clone MGC:52636 IMAGE:4681379), *c-myc* (Bugner et al., 2011), *celf1 (CUGBP Elav-like family, member 1)* (Day and Beck, 2011), *cryba1 (crystallin beta A1)* (Day and Beck, 2011), *egr2 (early growth response 2)* (Cizelsky et al., 2013), *foxc1 (forkhead box C1)* (Köster et al., 1998), *gata2 (gata binding protein 2)* (cDNA clone MGC:131004 IMAGE:7978680), *otx2 (orthodenticle homeobox 2)* (Lamb et al., 1993), *pax6* *(paired box 6t)* (Hitchcock et al., 1996; Hollemann et al., 1998), *pou4f1* (POU class 4 homeobox 1) (Liu et al., 2000), prox1 *(prospero homeobox 1)* (Dyer et al., 2003), *rax (retina and anterior neural fold homeobox)* (Furukawa et al., 1997), *rho (rhodopsin)* (Chang and Harris, 1998), *snai2 (snail family zinc finger 2)* (clone ID: pMX363), *sox3 (sex determining region Y-box 3)* (Maurus et al., 2005), *twist1 (twist family bHLH transcription factor 1)* (Gessert et al., 2007), and *vsx1 (visual system homeobox 1)* (Hayashi et al., 2000).

### Histology

Wildtype embryos as well as MO-injected embryos were embedded into gelatine and glutaraldehyde. Sections were performed with a thickness of 25 µm using a vibratome (Vibratome 1500 Classic, The Vibratome Company).

### Cartilage Staining by Alcian Blue Staining

In order to investigate the craniofacial cartilage, wildtype embryos and embryos injected with 20 ng Rpl5 MO were fixed at stage 45 and stained with Alcian blue as previously described (Gessert et al., 2007). Afterwards, the cranial cartilage was dissected and photographed.

### Phospho Histone 3 Staining and Terminal Deoxynucleotidyl Transferase dUTP Nick End Labeling Assay

Proliferative cells were stained for phospho histone 3 (pH3). Apoptotic cells were stained with the terminal deoxynucleotidyl transferase dUTP-biotin nick end labeling (TUNEL) assay. Both assays were performed at stage 13 and 23 according to established protocols (Gessert et al., 2007; Cizelsky et al., 2013).

### Quantitative Tissue Measurements

All quantitative measurements were performed using pictures of unilaterally Control MO, Rpl5 MO, Rpl5 MO + *rpl5* RNA, Rpl5 MO + *c-myc* RNA, Rpl5 MO + Tp53 MO, Rpl5 MO + *hBCL2* RNA -injected embryos of one representative experiment. The area of the eye, the apex angle of coloboma, and the head width were measured using the software ImageJ (Wayne Rasband). For brain size analyses, brains of fixed stage 42 embryos were dissected and photographed. ImageJ was used to measure the area of the brain.

To analyze the area of *tp53* and *c-myc* expression, Rpl5 MO and Control MO-injected embryos were photographed after WMISH. By using ImageJ, area of expression was selected and measured (Figures 6G,H, 7G,H, red area).

### RT-PCR

Total RNA was isolated from *Xenopus* embryos using the peqGOLD RNAPure Kit (PEQLAB) following the manufacturer´s protocol. cDNA synthesis was carried out using random primers and the Superscript II reverse transcriptase (Invitrogen). For semi-quantitative RT-PCR the following primers were used: *gapdh_RT_forward*: 5′-GCC GTG TAT GTG GTG GAA TCT-3′, *gapdh_RT_reverse*: 5′-AAG TTG TCG TTG ATG ACC TTT GC-3′, *rpl5_RT_forward*: 5′- GGT GCC TTC ACA TGC TAC CT-3′, and *rpl5_RT_reverse*: 5′- GCA CTG GAT TCT CCC GAA TA-3′.

### Imaging

For imaging whole *Xenopus* embryos, an Olympus MVX10 (fluorescence) or Olympus SZX12 microscope and an Olympus UC50 camera were used. Vibratome sections were imaged with an Olympus BX60 microscope and an Olympus DP70 or an Olympus DP28 camera. Images were processed with ImageJ and Affinity Designer 1.10.4.

### Statistics

Data was analyzed with the software GraphPad Prism 9. Only experiments with a higher survival rate than 50% and an absolute survival number of at least 20 individuals per group were considered for statistic evaluation. Only experiments with more than three independent experiments were evaluated statistically. To determine statistical differences the nonparametric Mann-Whitney rank sum test was used. Statistical significances are indicated as: *p ≤ 0.05; **p ≤ 0.01, ***p ≤ 0.001, ****p ≤ 0.0001.

## Results

### Genomic Analysis of *rpl5*

To compare the genomic region of *rpl5* between different species, an *in silico* synteny analysis was carried out. The genomes of *Homo sapiens*, *Mus musculus*, *Gallus gallus*, *Xenopus laevis* (both pseudoallels), *Xenopus tropicalis*, and *Danio rerio* were considered (Supplementary Figure S1A). The synteny analysis revealed a highly conserved genomic region of *rpl5*. Additionally, a protein alignment was performed, which showed high homology between the different species (Supplementary Figure S1B).

### *Ribosomal Protein L5* is Specifically Expressed in the Developing *Xenopus laevis*

**FIGURE 1:**
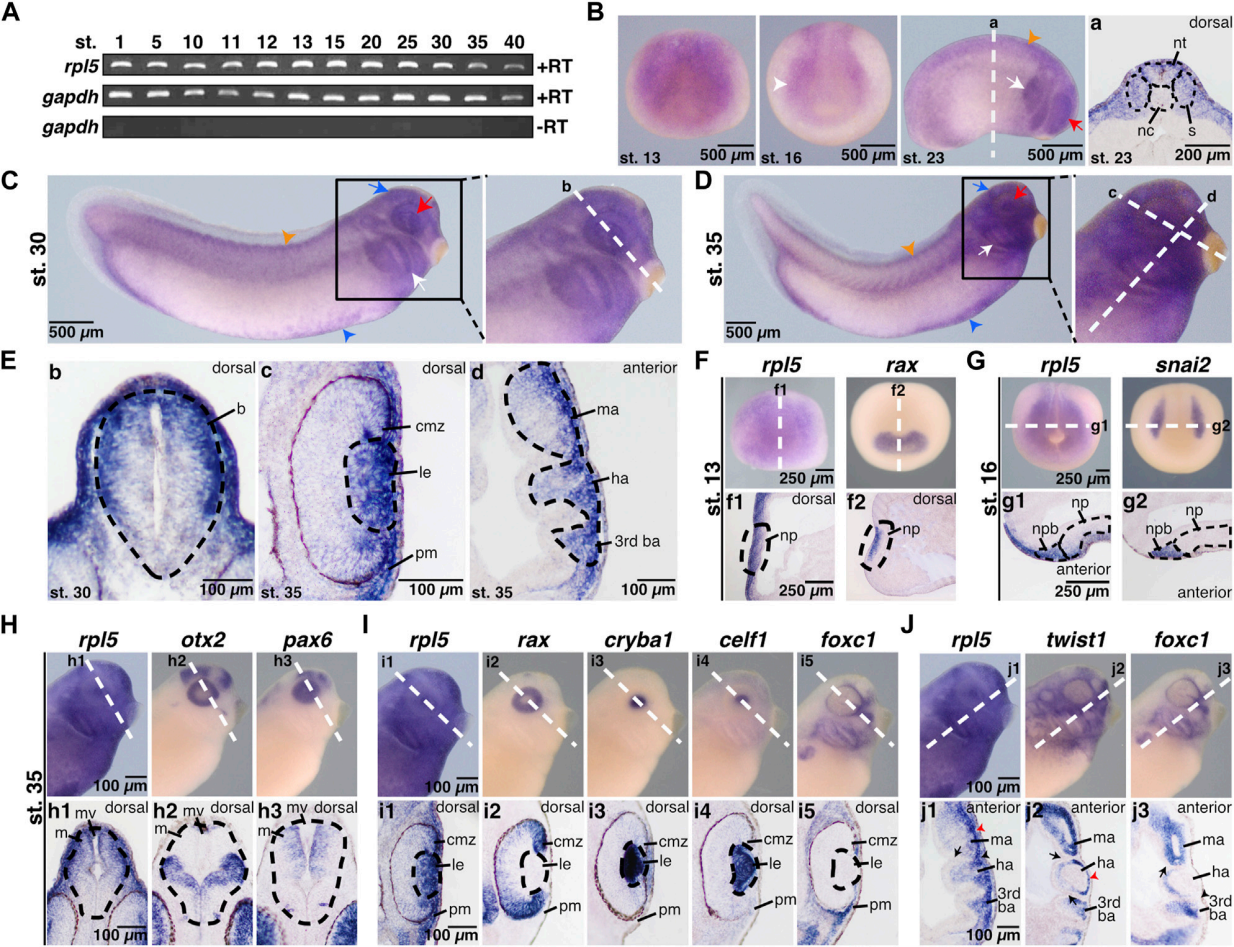
Spatial and temporal expression of *rpl5* in *Xenopus* laevis. **(A)** By reverse transcriptase PCR *rpl5* expression was analyzed in entire embryos throughout different developmental embryonic stages (stages 1–40). *rpl5* was found to be maternally (stages 1 and 5) and zygotically (stages 10–40) expressed during the entire embryonic development. *gapdh* served as loading control, *gapdh* minus reverse transcriptase as negative control. **(B–J)** *rpl5* expression and expression of different marker genes were visualized by whole mount *in situ* hybridization in *Xenopus laevis* at indicated stages. Section orientation is indicated in each figure by “dorsal” or “anterior” for dorsal-vegetal or anterior-posterior orientation, respectively. White dotted lines represent level of sections shown in lowercase letters **(a-j3)**. **(B)** Anterior views are shown for stage 13 and 16. During neurulation at stages 13 and 16, *rpl5* was mainly detected in the anterior neural plate (white arrowhead). At stage 23 (lateral view), *rpl5* was expressed in the branchial arches (white arrow), somites (orange arrowhead), and the developing eye (red arrow). **(a)** Transversal sections show *rpl5* expression in the neural tube and somites (indicated by black dotted lines), and the lateral mesoderm. **(C,D)** Lateral views. During late tailbud stages 30 and 35, *rpl5* expression was mainly detected in the developing eye (red arrow), the branchial arches (white arrow), the brain (blue arrow), the somites (orange arrowhead), and the ventral blood islands (blue arrowhead). **(E) (b)** Transversal section reveals *rpl5* expression in the brain (outlined by black dotted line), in which *rpl5* is enriched in the dorsal-lateral part of the mesencephalon. **(c)** Transversal section at stage 35, *rpl5* transcripts are found in the lens (outlined by black dotted line), the ciliary marginal zone and the periocular mesenchyme. **(d)** Longitudinal section is given. *rpl5* was expressed in the mandibular, hyoid and third branchial arch (outlined by black dotted line). **(F)** *rpl5* expression in comparison to the marker gene *rax* at stage 13. Anterior view of embryos is shown. Sagittal sections shown in **(f1)** and **(f2)** reveal expression of both genes in the neural plate (outlined by black dotted lines). **(G)** Expression of *rpl5* and *snai2* (marker gene for NCCs) at stage 16. Anterior views are given. During NCC induction, *rpl5* transcripts are enriched in the neural plate border (outlined by black dotted lines). **(g1)** and **(g2)** transversal sections are shown. **(H)** Expression of *rpl5* and the two brain marker genes *otx2* and *pax6* at stage 35. Lateral views are given. *rpl5* expression is mainly enriched in the dorsal part of the mesencephalon as shown in comparison to *otx2* and *pax6* expression. Brains are indicated by black dotted lines. **(h1–h3)** show transversal sections. **(I)** Expression of *rpl5* and the four marker genes *rax*, *cryba1*, *celf1*, and *foxc1*. Lateral views are shown. Transversal sections are given in **(i1–i5)**. *rpl5* expression is found in the ciliary marginal zone, where *rax* is expressed as well **(i1,2)**. *rpl5* transcripts are enriched in the lens (indicated by black dotted lines) as seen in comparison to the lens-specific marker genes *cryba* and *celf1* (i1,3,4), and in the NCC-derived periocular mesenchyme like *foxc1* **(i1,5)**. **(J)** Expression of *rpl5* and the two NCC marker genes *twist1* and *foxc1*. Lateral views are shown. **(j1–j3)** represent longitudinal sections. *rpl5* is expressed in all three branchial arches (mandibular arch, hyoid arch, and third branchial arch), where *twist1* and *foxc1* expression is located as well. Cranial placodes and ganglia are indicated by black arrowheads; red arrowheads indicate migrating NCCs and black arrows indicate the endodermal part of the pharyngeal pouches. Abbreviations: b, brain; ba, branchial arch; cmz, ciliary marginal zone; ha, hyoid arch; le, lens; m, mesencephalon; ma, mandibular arch; mv, mesencephalic ventricle; nc, notochord; np, neural plate; npb, neural plate border; nt, neural tube; pm, periocular mesenchyme; RT, reverse transcriptase; s, somites.

Earlier, *rpl5* expression was shown in whole embryos at stages 27 and 32 during *Xenopus* development (Scholnick et al., 1997; Wischnewski et al., 2000). Session et al. provided RNA-sequencing data showing *rpl5* expression throughout the entire embryonic development (Session et al., 2016). To provide a more detailed expression study, we here investigated *rpl5* expression by RT-PCR and WMISH during many different embryonic developmental stages. RT-PCR showed *rpl5* to be expressed maternally and zygotically throughout stages 1–40 (Figure 1A). WMISH with an *rpl5*-specific antisense probe was performed at various developmental stages starting after gastrulation throughout late tailbud stages to investigate the spatiotemporal expression of *rpl5* (Figures 1B–J). During stage 13 *rpl5* expression was mainly found in the anterior neural plate where *rax* is expressed as well (Figures 1B,F). At neural stages, *rpl5* is mainly expressed in the neural folds (white arrowhead), which was confirmed by *snai2* expression in whole mount embryos as well as sections (Figures 1B,G). At stage 23, *rpl5* expression was found in the migrating anterior neural crest cells (NCCs) (white arrow), the developing eye (red arrow), the neural tube, and the somites (orange arrowhead) as shown in comparison to the expression of somite-specific marker gene *actc1* (Figure 1B, Supplementary Figure S2A). At stage 28, *rpl5* expression is enriched in the ventral blood islands, where *gata2* and *hba3* are expressed as well (Supplementary Figure S2B). Late tailbud stages 30 and 35 show enriched *rpl5* expression in the developing eye (red arrow), the brain (blue arrow), the anterior NCCs (white arrow), the somites (orange arrowhead), and the ventral blood islands (blue arrowhead) (Figures 1C,D). Transversal sections of the midbrain at stage 30 and 35 revealed a stronger *rpl5* expression in the dorsal midbrain compared to the ventral part (Figures 1E,H); *otx2* and *pax6* were used as marker genes of the brain. Analyzing *rpl5* expression in the eye area at stage 35 showed strong expression in the lens and the ciliary marginal zone (CMZ) (Figures 1E,I). *rax* served as marker gene for the CMZ, *cryba1* and *celf1* as marker genes for the lens (Figures 1E,I). Furthermore, *rpl5* transcripts are enriched in the NCC-derived periocular mesenchyme as shown in comparison to *foxc1* expression (Figures 1E,I). In longitudinal sections of stage 35 embryos, *rpl5* expression is located in the mandibular arch, the hyoid arch and the third branchial arch, where *twist1* and *foxc1* are expressed as well (Figures 1E,J).

### Knockdown of Ribosomal Protein L5 Affects Proper *Xenopus* Eye Development

As Rpl5 was found to be highly expressed in anterior neural tissue, the impact of Rpl5 knockdown on anterior neural development of *Xenopus laevis* was investigated using an antisense-based MO approach. Therefore, an Rpl5 MO targeting the 5′UTR of *rpl5* was designed (Supplementary Figure S3A). Its binding affinity was checked with an MO binding affinity assay (Supplementary Figure S3B).

**FIGURE 2:**
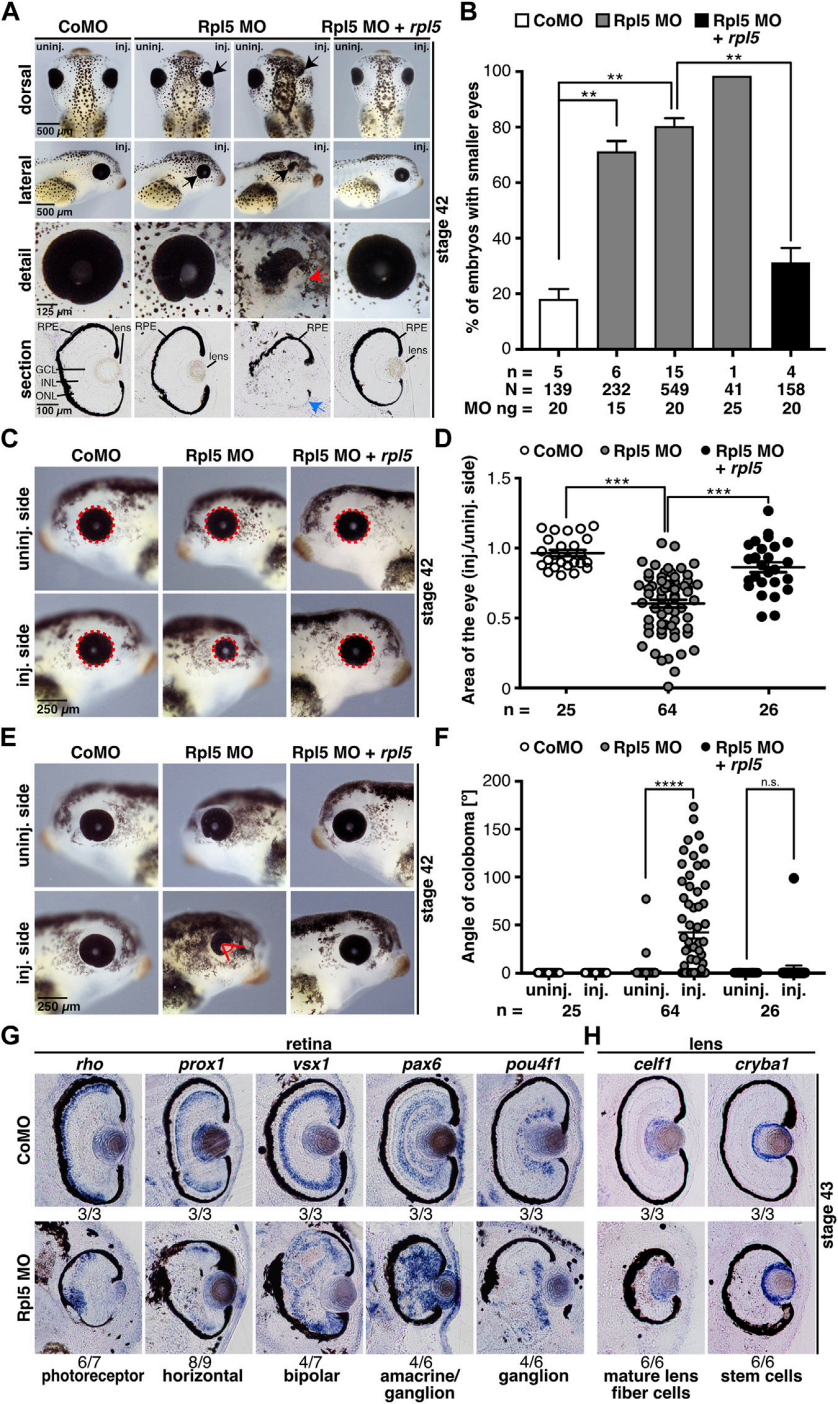
Rpl5 MO injection leads to a severe eye phenotype. **(A)** *Xenopus* embryos injected with Control MO, Rpl5 MO and Rpl5 MO + *rpl5* and evaluated at stage 42. The injected side was compared to the uninjected side. Dorsal, lateral, and detail views, as well as sections are shown. Rpl5 MO-injected embryos developed smaller and malformed eyes (black arrows) in a MO dose-dependent manner. Detailed view of deformed eyes is depicted (red arrow). Section view shows disrupted retinal pigmented epithelium (blue arrow, section orientation is dorsal (upper part) to ventral (lower part)). **(B)** Statistical analysis of data given in **(A)**. The eye phenotype was rescued by co-injecting 0.5 ng *rpl5* RNA. **(C)** The area of the eye (red dotted line) on the injected side was compared to uninjected side of Control MO, Rpl5 MO, and Rpl5 MO + *rpl5-*injected embryos at stage 42. **(D)** Statistical evaluation of data given in **(C)**. Embryos showed significantly smaller eyes upon Rpl5 depletion. This phenotype was rescued upon co-injection of *rpl5* RNA. **(E)** The angle of eye fissure (red lines) of stage 42 embryos was measured and the injected side compared to the uninjected side. **(F)** Statistical analysis of data given in **(E)**. Rpl5 morphants showed a coloboma phenotype in a large number of individuals. This phenotype was rescued upon co-injecting *rpl5* RNA. **(G)** At stage 43, different cell layers of the retinal lamination were analyzed by whole mount *in situ* hybridization using well-known marker genes as described in the main text. Transversal sections of Control MO and Rpl5 MO-injected embryos are depicted. Upon Rpl5 knockdown all depicted cell types were delocalized and cell layers are disrupted. Number below the columns indicate the number of embryos showing the depicted phenotype per number of embryos analyzed. Section orientation is dorsal (upper part) to ventral (lower part). **(H)** *celf1*, a marker gene for mature lens fiber cells and *cryba1*, a marker gene for lens stem cells, were analyzed at stage 43 embryos injected with either Rpl5 MO or Control MO. Both lens-specific marker genes were not affected upon Rpl5 MO injection. Number below the columns indicate the number of embryos showing the depicted phenotype per number of embryos analyzed. Section orientation is dorsal (upper part) to ventral (lower part). Abbreviations: CoMO, Control morpholino oligonucleotide; GCL, ganglion cell layer; INL, inner nuclear cell layer, inj., injected; n, number of independent experiments; N, number of injected embryos and analyzed; n.s., non-significant; ONL, outer nuclear cell layer; RPE, retinal pigmented epithelium; Rpl5 MO, ribosomal protein L5 morpholino oligonucleotide; uninj., uninjected. Error bars indicate standard error of the means; **p ≤ 0.01, ***p ≤ 0.001, ****p ≤ 0.0001.

To reduce Rpl5 translation, the MO was injected into one animal-dorsal blastomere of eight-cell stage embryos to directly target anterior neural tissue (Moody and Kline, 1990). The uninjected site served as internal control and co-injection of 0.5 ng *GFP* RNA was used to track the correct injection site. Control MO injections served as injection control. Injection of Rpl5 MO led to deformed and smaller eyes in a MO dose-dependent manner whereas Control MO injection led to normally developed eyes (Figures 2A,B). Additionally, vibratome sections showed a disturbed retinal pigmented epithelium (RPE) (Figure 2A). To describe the observed eye defects in more detail, we quantitatively measured the area of the eye on the injected and uninjected side, respectively. The injection of Rpl5 MO led to a reduction of the eye area of around 40% compared to the uninjected side and to Control MO injected embryos (Figures 2C,D). Furthermore, we investigated the formation of colobomas by measuring the apex angle. The injection of Rpl5 MO resulted in a coloboma phenotype (Figures 2E,F). All above-described eye phenotypes were rescued upon co-injection of Rpl5 MO together with an *rpl5* RNA, which is not targeted by the Rpl5 MO, implicating the specificity of the Rpl5 MO-induced eye phenotype (Figures 2A–F).

To further analyze the disturbed RPE upon Rpl5 MO injection, WMISH experiments were performed using well-known retina cell type marker genes (Cizelsky et al., 2013): *rho* for photoreceptor cells, *prox1* for horizontal cells, *vsx1* for bipolar cells, *pax6* for amacrine and ganglion cells, and *pou4f1* for ganglion cells. Vibratome sections showed a severe disorganization of all different retinal layers whereas the uninjected side and Control MO-injected embryos showed proper retinal organization (Figure 2G and Supplementary Figure S4). As Rpl5 was found to be strongly expressed in the lens (Figure 1E), we analyzed two lens-specific marker genes, *celf1* for mature lens fiber cells and *cryba1* for lens stem cells (Day and Beck, 2011). Neither the injection of Rpl5 MO nor Control MO affected the lens-specific marker genes (Figure 2H).

**FIGURE 3:**
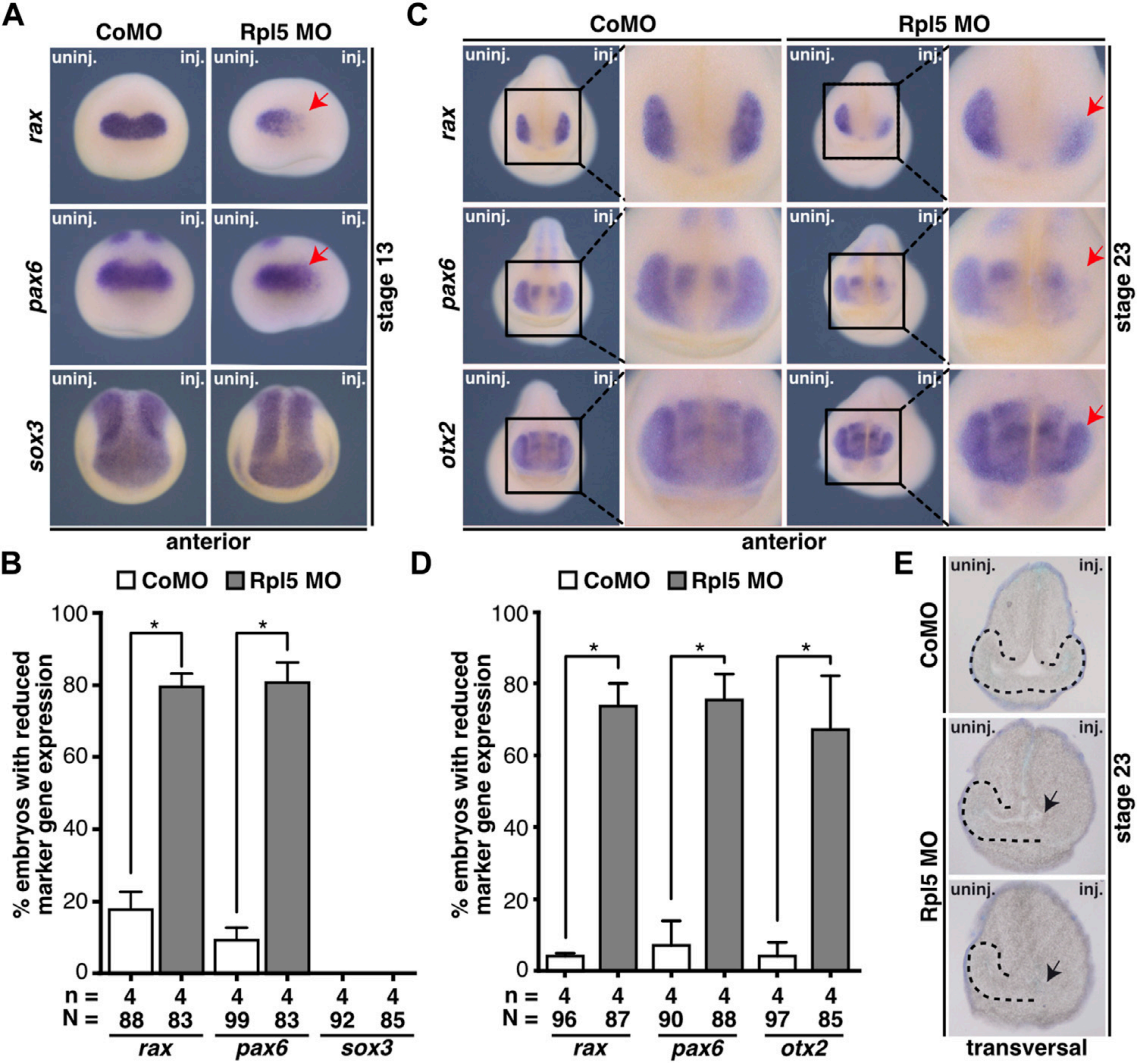
Rpl5 knockdown interferes with eye-specific marker expression and eye vesicle evagination. **(A)** Anterior views of stage 13 embryos are given. By whole mount *in situ* hybridization the expression of the eye-specific marker genes *rax* and *pax6* and the pan-neural marker gene *sox3* was investigated in embryos injected with Control MO or Rpl5 MO. The expression of *rax* as well as *pax6* was reduced upon Rpl5 depletion (red arrows), whereas Control MO injection did not alter the expression. Expression of *sox3* was not altered, neither in Control MO nor in Rpl5 MO injected embryos. **(B)** Statistical analysis of data shown in **(A)**. **(C)** Anterior views are depicted. At stage 23, Control MO and Rpl5 MO-injected embryos were analyzed regarding the expression of eye-specific marker genes *rax*, *pax6*, and *otx2* by whole mount *in situ* hybridization. All three marker genes showed reduced expression in the eye field (red arrows) upon Rpl5 knockdown. Control MO injection did not result in altered gene expression. **(D)** Statistical analysis showed a significantly reduced expression in all three marker genes. **(E)** Transversal sections of stage 23 embryos injected with either Control MO or Rpl5 MO are shown. Section orientation is dorsal (upper part) to ventral (lower part). Eye vesicles in embryos injected with Rpl5 MO do not evaginate (indicated by black arrows). Control MO injection do not affect eye vesicle evagination. Eye vesicle is indicated by black dotted line. Abbreviations: CoMO, Control morpholino oligonucleotide; inj., injected; n, number of independent experiments; N, number of injected embryos and analyzed; Rpl5 MO, ribosomal protein L5 morpholino oligonucleotide; uninj., uninjected. Error bars indicate standard error of the means; *p ≤ 0.05.

Additionally, the expression of eye-specific marker genes was analyzed *via* WMISH upon Rpl5 reduction to investigate the molecular basis of the described eye phenotype (Figures 3A–D). During eye field induction at stage 13, the expression of the eye-specific marker genes *rax* and *pax6* were significantly reduced upon Rpl5 MO injection, whereas the Control MO injection had no effect on both marker genes. The pan-neural marker gene *sox3* was not affected upon Rpl5 as well as Control MO injection (Figures 3A,B). At stage 23, when eye cells differentiate, the expression of *rax*, *pax6*, and *otx2* was reduced in around 70% of the Rpl5 morphants, whereas Control MO-injected embryos showed normally expressed marker genes (Figures 3C,D).

Since the expression of eye-specific marker genes was affected in Rpl5 morphants, we further analyzed the eye tissue in sections of Rpl5 MO and Control MO-injected embryos. Rpl5 depletion led to a disturbed evagination of the eye vesicle on the injected side. Control MO injection had no effect on eye vesicle development (Figure 3E).

### Affected Brain Development Upon Knockdown of Ribosomal Protein L5

**FIGURE 4:**
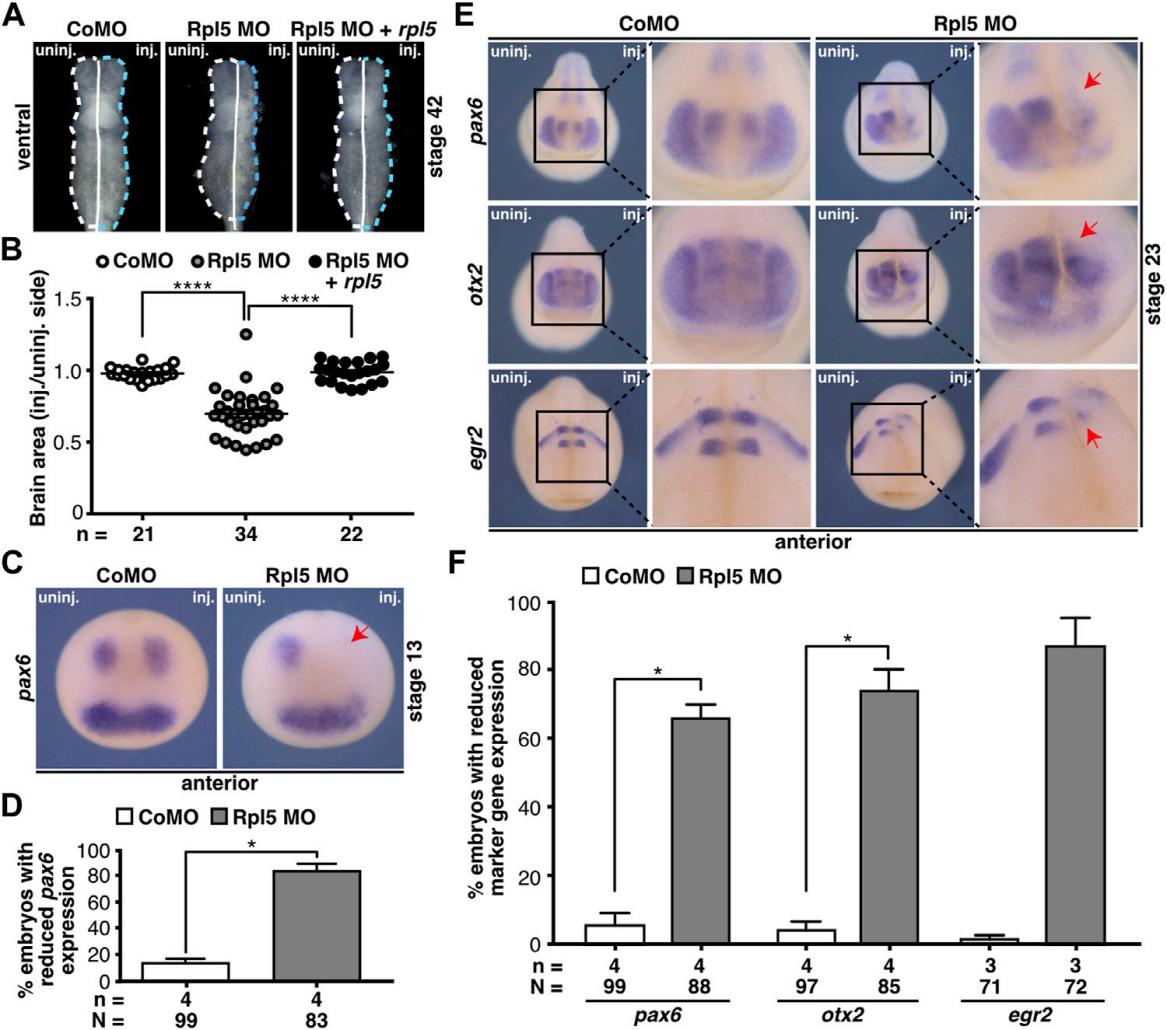
Rpl5 depletion affects proper brain development. **(A)** Brains of Control MO, Rpl5 MO, and Rpl5 MO + *rpl5* RNA-injected embryos were dissected, the area of the brains was measured, and injected side was compared to uninjected side. Dotted lines indicate measured area. Ventral views of brains are given. **(B)** The area of the Rpl5 MO-injected side was significantly smaller compared to the uninjected side. Control MO-injection did not affect the area of the brain. This phenotype was rescued upon co-injecting *rpl5* RNA. **(C)** Anterior views of embryos are depicted. The marker gene *pax6* was analyzed in the anterior neural tube of embryos injected either with Control MO or Rpl5 MO. Rpl5 morphants showed a drastic reduction in expression (red arrow) on the injected side in 80% of the embryos, whereas Control MO injected embryos showed no reduction in *pax6* expression. **(D)** Statistical analysis of data shown in **(C)**. **(E)** Anterior views of embryos are given. At stage 23, the brain-specific marker genes *pax6*, *otx2*, and *egr2* were investigated by whole mount *in situ* hybridization in embryos injected with Control MO or Rpl5 MO. In the developing brain, all three investigated marker genes showed reduced expression (red arrows) upon Rpl5 MO injection but not upon Control MO injection. **(F)** Statistical analysis of data shown in **(E)**. Abbreviations: CoMO, Control morpholino oligonucleotide; inj., injected; n, number of independent experiments; N, number of injected embryos and analyzed; Rpl5 MO, ribosomal protein L5 morpholino oligonucleotide; uninj., uninjected. Error bars indicate standard error of the means; *p ≤ 0.05, ****p ≤ 0.0001.

The impact of Rpl5 depletion was further investigated in the brain (Figure 4). Therefore, brains from Rpl5 MO- as well as Control MO-injected embryos fixed at stage 42 were isolated. Area measurements of both hemispheres showed a significant reduction of the brain upon Rpl5 knockdown. The uninjected side, as well as Control MO-injected embryos showed no reduction. Furthermore, this phenotype was rescued upon co-injecting *rpl5* RNA (Figures 4A,B).

In order to gain further insights into the molecular basis, we performed WMISH experiments using well-established brain marker genes: *pax6* for the forebrain, *otx2* for the forebrain and midbrain, and *egr2* for the hindbrain. At stage 13, embryos injected with Rpl5 MO showed a drastic decrease in *pax6* expression in the neural plate (Figures 4C,D). The expression of all three marker genes, *pax6, otx2,* and *egr2,* was reduced during brain cell differentiation at stage 23 at the Rpl5 MO-injected side (Figures 4E,F). Brain marker gene expression was not affected at the uninjected side as well as in Control MO-injected morphants.

### Ribosomal Protein L5 Interferes With Cranial Cartilage Development in *Xenopus*

**FIGURE 5:**
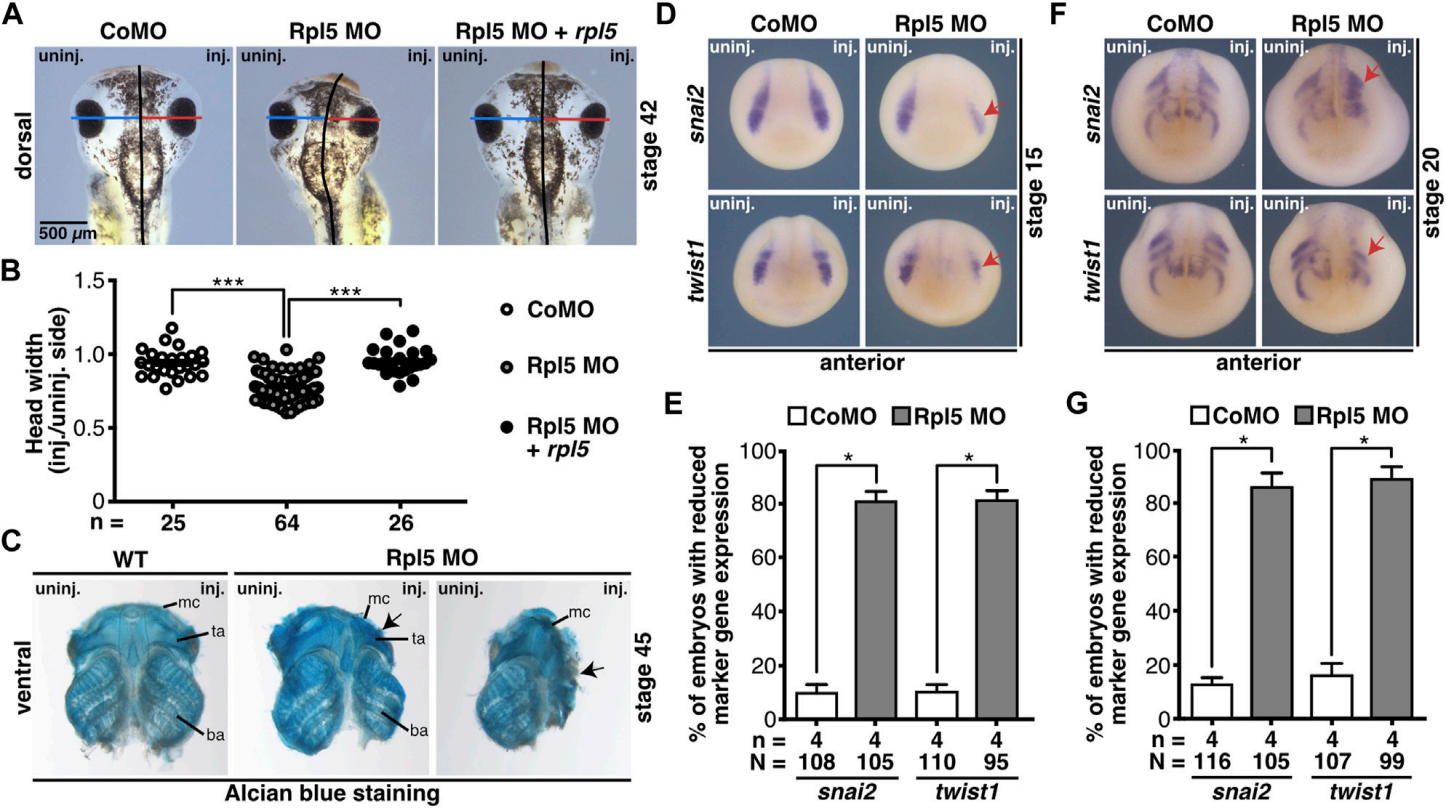
Rpl5 MO injection hinders proper development of the cranial cartilage. **(A)** The head width was measured and the Control MO, Rpl5 MO, or Rpl5 MO + *rpl5* RNA-injected side compared to the uninjected side of stage 42 embryos. Blue and red lines indicate measured width; black line represents the embryo midline. **(B)** Rpl5 depletion led to significantly narrower heads, which was rescued upon *rpl5* RNA co-injecting. **(C)** Cranial cartilage was dissected from stage 45 wildtype embryos and Rpl5 morphants. Rpl5 knockdown resulted in deformed cartilages structures (black arrows) like the Meckel’s cartilage, the tectum anterius, and the branchial arch. Ventral views of cranial cartilages are shown. **(D)** Anterior views of embryos are depicted. *snai2* and *twist1* were investigated as marker genes of the neural crest in stage 15 embryos. Reduced marker gene expression is indicated with red arrows. **(E)** Upon Rpl5 depletion both marker genes were significantly reduced in 80% of the embryos. **(F)** Anterior views of embryos are given. Expression of *snai2* and *twist1* were analyzed at stage 20 during NCC migration. Red arrows indicate reduced marker gene expression. **(G)** Both marker genes showed a significant reduction in expression in around 85% of the Rpl5 MO-injected embryos. Abbreviations: ba, branchial arch; CoMO, Control morpholino oligonucleotide; inj., injected; mc, Meckel’s cartilage; n, number of independent experiments; N, number of injected embryos and analyzed; Rpl5 MO, ribosomal protein L5 morpholino oligonucleotide; ta, tectum anterius; uninj., uninjected; WT, wildtype. Error bars indicate standard error of the means; *p ≤ 0.05, ***p ≤ 0.001.

As shown in Figure 1, *rpl5* is highly expressed in the anterior NCCs which contribute to the formation of the cranial cartilage (Jacobson, 1991). Therefore, head development including cranial cartilage structures was investigated upon Rpl5 knockdown. Head width measurements showed a significantly narrower head on the Rpl5-MO-injected side, whereas Control MO injection resulted in normally developed heads (Figures 5A,B). This phenotype was rescued by co-injecting *rpl5* RNA. Furthermore, cranial cartilages of MO-injected embryos were stained by Alcian blue and dissected at stage 45 to accurately describe the observed phenotype. Structures of the cranial cartilage such as the Meckel’s cartilage, the tectum anterius, and the branchial arches were reduced and disrupted upon Rpl5 depletion (Figure 5C). The wildtype control as well as the uninjected side showed normally developed cranial cartilages.

To analyze the molecular basis of the described phenotype, the NCC-specific marker genes *snai2* and *twist1* were investigated by WMISH experiments at stages 15 and 20. During NCC induction (stage 15), both marker genes showed a decrease in expression in around 80% of the Rpl5 MO-injected embryos. During NCC migration (stage 20) *snai2* as well as *twist1* expression was reduced and shortened in 80–90% of the Rpl5 MO- compared to Control MO-injected embryos (Figures 5D–G).

### Loss of Ribosomal Protein L5 Affects *Xenopus* Blood Development

Rpl5 was also found to be expressed in the blood islands (Figure 1, Supplementary Figure S2B). To investigate Rpl5 depletion in this disease-relevant tissue, we analyzed two hematopoiesis specific marker genes, *gata2* and *hba3*. *gata2* is a hematopoietic transcription factor and serves as marker gene for hematopoietic stem and progenitor cells (Katsumura et al., 2017), whereas *hba3* is part of hemoglobin and therefore located in mature erythrocytes. *gata2* expression was not affected upon Rpl5 depletion (Supplementary Figures S5A,B). However, the expression of *hba3* was reduced on the Rpl5 MO-injected side (Supplementary Figures S5C,D). Control MO injection did not alter expression of either of the marker genes.

Taken together, Rpl5 is crucial for a proper development of the eye, the brain, the NCC-derived cranial cartilage, and the blood during *Xenopus* embryogenesis. The observed phenotypes in *Xenopus laevis* mirror the clinical manifestations seen in patients suffering from ribosomopathies, which makes *Xenopus* a valuable model organism to investigate the disease in detail.

### Rpl5 Depletion Affects Proliferative Pathways

**FIGURE 6:**
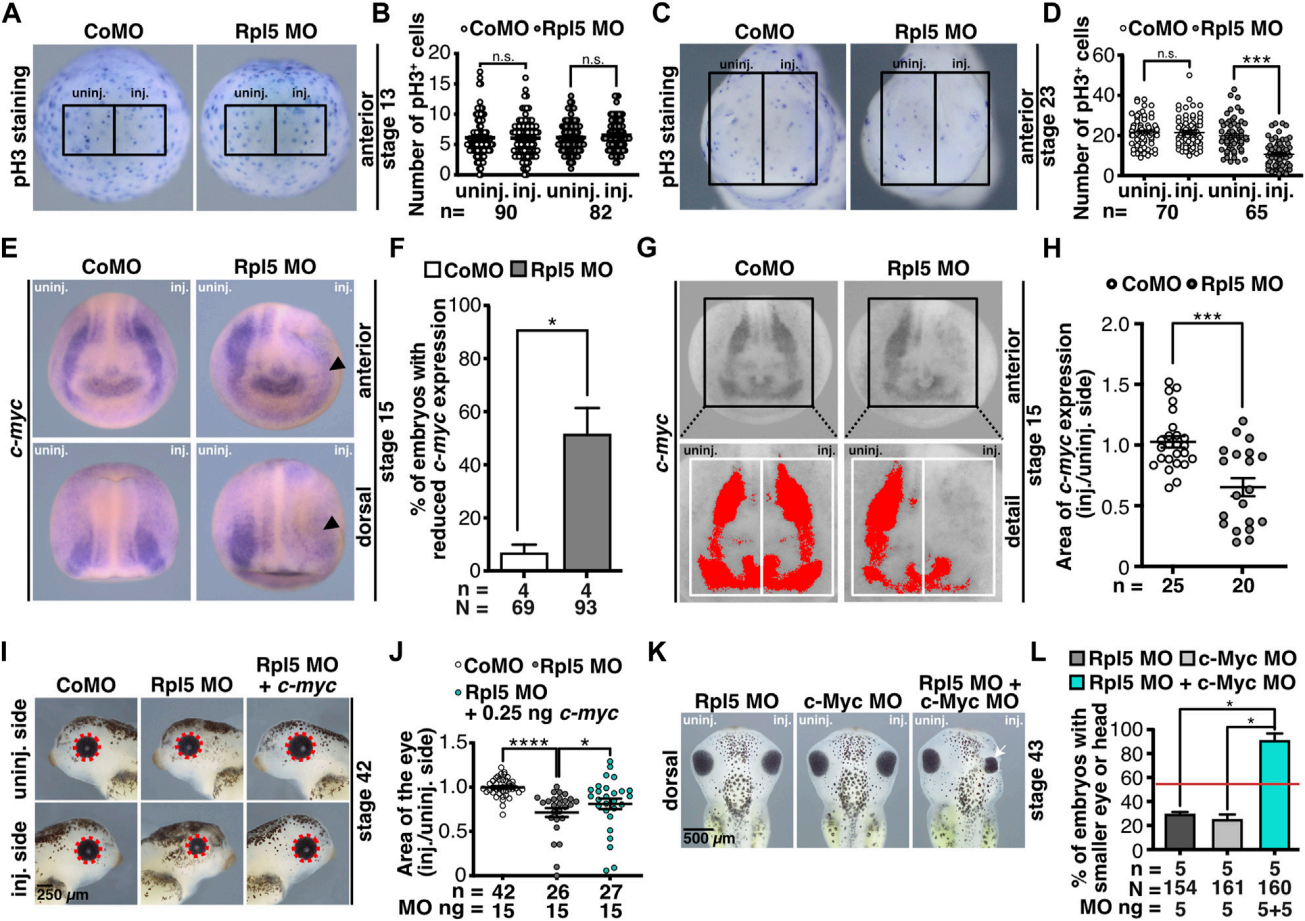
Rpl5 depletion interferes with pathway of proliferation. **(A)** Proliferative cells of embryos injected with Control MO or Rpl5 MO were stained *via* a pH3 antibody at stage 13. Anterior views are shown. Number of proliferative cells were compared in the area of the anterior neural plate of the injected side and the uninjected side (black boxes indicate the area where proliferative cells were counted). **(B)** In Rpl5 morphants and control embryos the number of pH3 positive cells did not alter between injected and uninjected side. **(C)** At stage 23 proliferative cells were stained *via* pH3 staining in Rpl5 and Control MO injected embryos. Anterior view of embryos is shown. The number of proliferative cells was compared between injected and uninjected side (black boxes indicate the area where cells were counted). **(D)** Rpl5 morphants had a reduced number of pH3 positive cells on the injected side, whereas the uninjected side and Control MO-injected embryos were not affected. **(E)** Embryos injected with either Control MO or Rpl5 MO were fixed at stage 15 and *c-myc* expression was analyzed by WMISH. Reduced *c-myc* expression is indicated by black arrowhead. **(F)** *c-myc* expression was significantly reduced on the Rpl5 MO-injected side, whereas Control MO injection did not alter *c-myc* expression. **(G)** Area of *c-myc* expression (red area) was measured in stage 15 embryos injected with either Control MO or Rpl5 MO. The injected side was compared to the uninjected side (white boxes). **(H)** Upon Rpl5 depletion, the area of *c-myc* expression was significantly reduced in Rpl5 morphants compared to control embryos. **(I)** Embryos were injected with Control MO, Rpl5 MO, or Rpl5 MO + 0.25 ng *c-myc* RNA, fixed at stage 42 and photographed. The area of the eye (red dotted line) was measured and the injected side was compared to the uninjected side. **(J)** 15 ng Rpl5 MO injection resulted in significantly smaller eyes. Injection of 15 ng Rpl5 MO together with 0.25 ng *c-myc* RNA significantly increased the eye area compared to Rpl5 MO injection alone. Control MO injection did not affect the eye area. **(K)** Eight-cell stage embryos were injected with 10 ng Control MO, 5 ng Rpl5 MO and 5 ng c-Myc MO alone or in combination. Embryos were fixed and analyzed regarding a smaller head or eye (white arrow) at stage 43. **(L)** Embryos injected with Control MO did not develop malformed eyes or heads. Rpl5 MO and c-Myc MO injection alone resulted in smaller eyes or narrower heads in around 30% of the embryos. The simultaneous injection of Rpl5 MO and c-Myc MO led to smaller eyes or heads in 90% of the embryos. Red line indicates sum of embryos showing an eye or head phenotype injected with 5 ng Rpl5 MO and 5 ng c-Myc MO. Abbreviations: *c-myc*, *Myc proto-oncogene*; CoMO, Control morpholino oligonucleotide; inj., injected; n, number of independent experiments; N, number of injected embryos and analyzed; n.s., non-significant; pH3, phospho histone 3; Rpl5 MO, ribosomal protein L5 morpholino oligonucleotide; uninj., uninjected. Error bars indicate standard error of the means; *p ≤ 0.05, ***p ≤ 0.001, ****p ≤ 0.0001.

To investigate whether the reduced anterior neural structures are a result of a disturbed cell proliferation, stage 13 and 23 embryos were analyzed *via* pH3 staining. At stage 13 the number of proliferative cells did not alter upon Rpl5 MO injection (Figures 6A,B). However, at stage 23 Rpl5 depletion resulted in a significantly decreased number of proliferative cells (Figures 6C,D). Control MO injection did not lead to altered proliferation (Figures 6A–D).

c-Myc is a crucial regulator for proliferative processes in the cell (Gomez-Roman et al., 2003; Arabi et al., 2005). Since the number of proliferative cells decreased in Rpl5-depleted embryos, we investigated c-Myc in the following experiments. Furthermore, *c-myc* RNA has been shown to be degraded by free Rpl5 and Rpl11 upon cellular stress, e.g., induced by ribosomal biogenesis defects as they occur during ribosomopathies (Liao et al., 2014). Additionally, the knockdown of c-Myc has been found to lead to a cranial cartilage phenotype in *Xenopus* embryos as well as mice (Bellmeyer et al., 2003; Wei et al., 2007) similar as observed here for Rpl5 depletion. Hence, *c-myc* expression was analyzed in stage 15 embryos injected with Rpl5 MO. The embryos showed a reduced *c-myc* expression on the injected side, whereas Control MO injection had no effect on *c-myc* expression (Figures 6E,F). A quantitative analysis confirmed this result (Figures 6G,H). Furthermore, we co-injected Rpl5 MO and *c-myc* RNA to analyze a possible rescue mechanism. The area of the eyes was quantitatively measured and injected side was compared to uninjected side. Upon co-injecting Rpl5 MO together with 0.25 ng *c-myc* RNA, the area of the injected eye significantly increased in comparison to Rpl5 MO injection (Figures 6I,J). To further investigate a common pathway of Rpl5 and c-Myc, low doses of Rpl5 MO and c-Myc MO were injected alone and in combination. Low doses of Rpl5 MO and c-Myc MO resulted in a mild eye or head phenotype in few embryos. The injection of both MOs together led to a more than additive eye or head phenotype in a high number of embryos (Figures 6K,L). This effect was not extended to the number of proliferative cells at stage 23. This was analyzed by pH3 staining in embryos injected with low doses of Rpl5 MO and c-Myc MO alone and in combination (Supplementary Figures S6A,B).

In conclusion, this data suggests that Rpl5 depletion affects proliferation during organogenesis. c-Myc and Rpl5 share a common pathway and rescue experiments indicate that c-Myc is downstream of Rpl5.

### Rpl5 Depletion Affects Apoptotic Pathways

**FIGURE 7:**
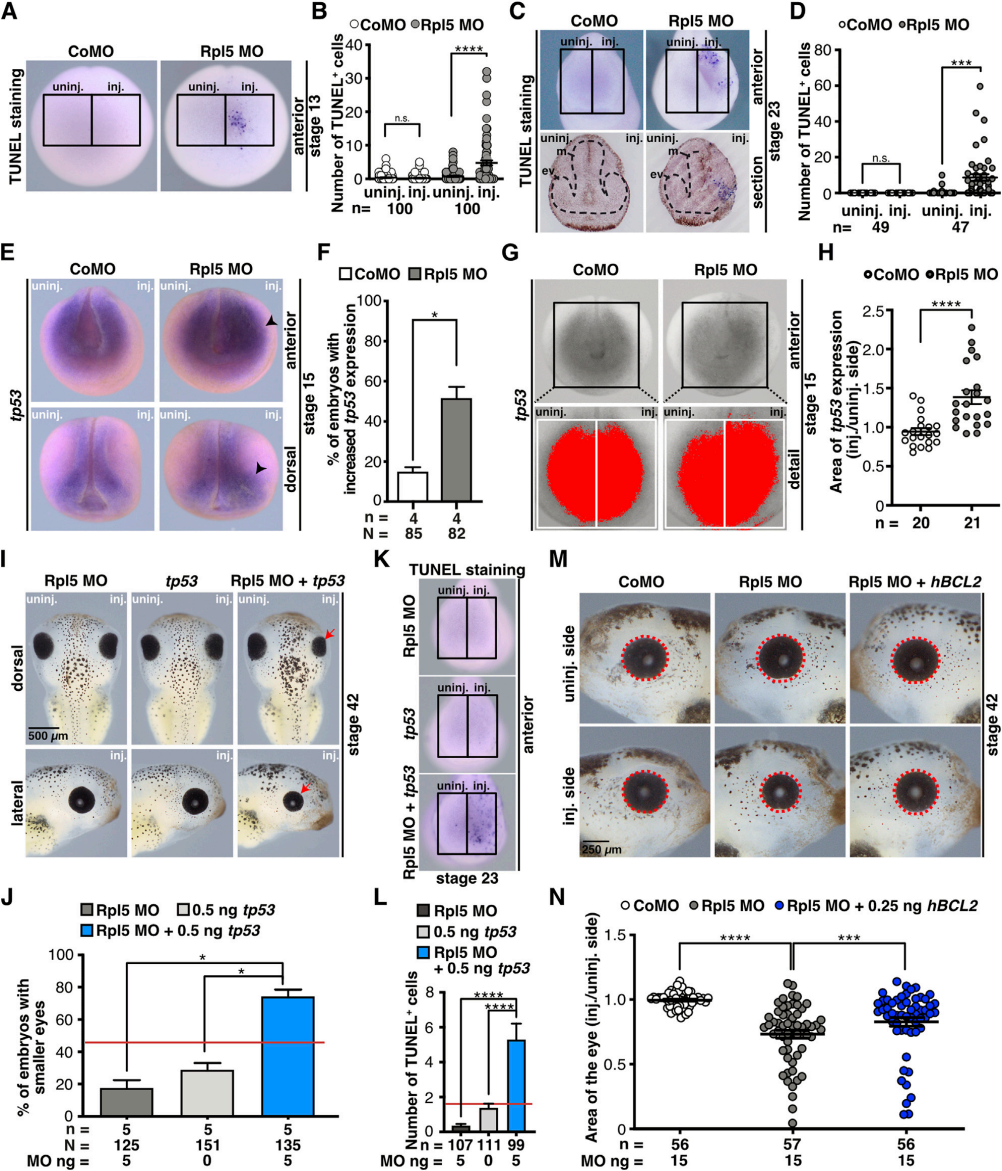
Rpl5 depletion affects apoptotic pathways. **(A)** Late apoptotic cells were stained in stage 13 embryos injected with Control and Rpl5 MO *via* TUNEL staining. Anterior views are shown. Number of apoptotic cells was counted in the area of the anterior neural plate (black boxes indicate the area where apoptotic cells were counted) and the injected side was compared to the uninjected side. **(B)** Control MO injection did not increase the number of apoptotic cells. Rpl5 MO injection led to a significantly increased number of apoptotic cells. **(C)** Anterior views are shown in the upper row. Lower row shows transversal sections of embryos heads [dorsal (upper part) to ventral (lower part) oriented section]. Black dotted line indicates eye vesicle and mesencephalon. Apoptotic cells were detected *via* TUNEL staining at stage 23 and counted in the anterior part of whole embryos (indicated by black boxes). The injected side was compared to the uninjected side. **(D)** Upon Rpl5 depletion, embryos depicted an increased number of apoptotic cells in the anterior neural tissue. **(E)** At stage 15, embryos injected with Control MO or Rpl5 MO were fixed and *tp53* expression was analyzed by WMISH. Anterior and dorsal views are given. Increased *tp53* expression is indicated by black arrowheads. **(F)** More than 50% of the embryos showed an increase in *tp53* expression upon Rpl5 knockdown. **(G)** The area of *tp53* expression (red area) on the injected side was compared to the uninjected side (white boxes) in stage 15 embryos. **(H)** Compared to the control group, the area of *tp53* increased significantly upon Rpl5 MO injection. **(I)** 5 ng Rpl5 MO and 0.5 ng *tp53* RNA were injected alone and in combination into eight-cell stage embryos. At stage 42, embryos were analyzed regarding a smaller eye phenotype (red arrows). Injection of Rpl5 MO or *tp53* RNA alone resulted in smaller eyes in a small number of embryos (18 and 30%). Injection of both resulted in smaller eyes in more than 70% of the embryos. **(J)** Statistical analysis of data given in **(I)**. Red line indicates sum of embryos showing an eye phenotype injected with 5 ng Rpl5 MO and 0.5 ng *tp53* RNA. **(K)** Embryos were injected with 5 ng Rpl5 MO or 0.5 ng *tp53* RNA alone or in combination, fixed at stage 23 and apoptotic cells were stained *via* TUNEL staining. Anterior view of embryos is given. The number of apoptotic cells was counted in the anterior region (indicated by black boxes). **(L)** Rpl5 MO and *tp53* RNA injection resulted in a low number of TUNEL positive cells. Combined injection shows a high number of TUNEL positive cells. Red line indicates sum of TUNEL positive cells in embryos injected with 5 ng Rpl5 MO and 0.5 ng *tp53* RNA. Data of TUNEL staining is depicted after background noise reduction, performed for each injection condition separately. This was achieved by subtracting the average number of TUNEL positive cells of the uninjected side of the nominal number of TUNEL positive cells of the injected side. If this resulted in a negative value, it is reported as zero. **(M)** At eight-cell stage, embryos were injected with Control MO, Rpl5 MO, or Rpl5 MO together with 0.25 ng *hBCL2* RNA and fixed at stage 42. Eye area (red dotted line) was quantitatively measured and injected side was compared to the uninjected side. **(N)** Control MO injection did not affect the area of the eye, whereas Rpl5 MO injection led to smaller eyes. The combined injection of Rpl5 MO and 0.25 ng *hBCL2* significantly increased the area of the eye compared to Rpl5 MO injection alone. Abbreviations: CoMO, Control morpholino oligonucleotide; ev, eye vesicle; *hBCL2*, *human B cell lymphoma 2*; inj., injected; m, mesencephalon; n, number of independent experiments; N, number of injected embryos and analyzed; n.s., non-significant; Rpl5 MO, ribosomal protein L5 morpholino oligonucleotide; *tp53*, *tumor protein p53*; TUNEL, Terminal deoxynucleotidyl transferase dUTP-biotin nick end labeling; uninj., uninjected. Error bars indicate standard error of the means; *p ≤ 0.05, ***p ≤ 0.001, ****p ≤ 0.0001.

To investigate whether cell apoptosis contributes to the observed anterior phenotypes, we analyzed stage 13 and stage 23 embryos *via* TUNEL staining upon Rpl5 depletion. At both developmental stages, the anterior tissue showed a significantly increased number of apoptotic cells on the injected side (Figures 7A–D). At stage 23, transversal sections of the head showed TUNEL-positive cells in the developing eye tissue (Figure 7C). Control MO injection did not increase the number of apoptotic cells at stage 13 or 23.

Previous studies described that upon defective ribosomal biogenesis—e.g., induced by Rpl5 knockdown—free Rpl5 or Rpl11 or the 5S-RNP complex can lead to Tp53 pathway activation by binding MDM2 and hence an apoptotic signalling cascade is activated (Dai and Lu, 2004; Sulima et al., 2019). Furthermore, it was shown in several animal models that the knockout of *tp53* rescued craniofacial phenotypes occurring during ribosomopathies (Jones et al., 2008; Zhao et al., 2014; Griffin et al., 2015; Calo et al., 2018). Therefore, we investigated whether the simultaneous knockdown of Rpl5, which itself might induce defective ribosomal biogenesis, and Tp53 can rescue the Rpl5 MO-induced phenotype. However, the phenotype was not rescued (Supplementary Figures S6C,D). To exclude also a subtle rescue, the eye area was analyzed quantitatively; which also showed no rescue (Supplementary Figures S6E–H). At stage 15, however, *tp53* expression was significantly increased on the Rpl5 MO-injected side, whereas the un-injected side or the Control MO injection did not alter *tp53* expression (Figures 7E,F). A quantitative analysis confirmed the *tp53* increase upon Rpl5 depletion (Figures 7G,H). To further characterize a possible common pathway between Rpl5 and Tp53, we investigated whether the gain of Tp53 function worsens the Rpl5 MO-induced phenotype by injecting Rpl5 MO and *tp53* RNA alone and in combination using low doses into one animal-dorsal blastomere of eight-cell stage embryos. The injection of either 5 ng Rpl5 MO or 0.5 ng *tp53* RNA resulted in a small number of embryos with smaller eyes (30 and 17% respectively). In contrast, the combined injection of both in low doses resulted in smaller eyes in around 75% of the injected embryos (Figures 7I,J). Furthermore, low doses of either Rpl5 MO or *tp53* RNA were injected alone or in combination into the animal-dorsal blastomere of eight-cell stage embryos and the number of apoptotic cells was analyzed at stage 23 *via* TUNEL staining. The injection of either Rpl5 MO or *tp53* RNA resulted in a small number of apoptotic cells on the injected side. However, the simultaneous injection of both low doses increased the number of apoptotic cells drastically (Figures 7K,L). This more than additive effect of Rpl5 and Tp53 on the late phenotype as well as the number of apoptotic cells at stage 23 suggests a common pathway of the two molecules.

To further investigate whether apoptosis contributes to the observed phenotypes, Rpl5 MO was injected in combination with the apoptosis blocker *hBCL2* RNA. BCL2 blocks the intrinsic apoptotic pathway by several mechanisms (Siddiqui et al., 2015). Rpl5 MO was injected alone and together with 0.25 ng *hBCL2* RNA unilaterally into one animal-dorsal blastomere of eight-cell stage embryos. At stage 42, the area of the eye was measured and the injected side was compared to the uninjected side. The co-injection of *hBCL2* RNA partially rescued the eye phenotype in Rpl5 morphants (Figures 7M,N).

## Discussion

In this study, the ribosomal protein Rpl5 has been shown to be required for proper development of anterior organs and tissues such as the eyes, the brain, and the cranial cartilage in *Xenopus laevis*. Furthermore, the Rpl5 knockdown led to increased apoptosis and decreased proliferation. These findings were in line with decreased *c-myc* expression and increased *tp53* expression. Additionally, common pathways were elucidated between Rpl5 and Tp53 and c-Myc, respectively. The co-injection of apoptosis blocker *BCL2* led to a partial rescue of the Rpl5 MO-induced eye phenotype. We found that Rpl5 depletion results in early phenotypes occurring before organogenesis—even though in previous studies early *Xenopus* development has been shown to be independent of ribosomal biogenesis (Brown, 1964; Pierandrei-Amaldi and Amaldi, 1994). This suggests that Rpl5 might have additional functions beyond its role in ribosome formation.

The extensive expression analysis of *rpl5* throughout the embryonic development of *Xenopus laevis* showed a detailed expression in the neural tissue, such as the developing eye and brain, as well as the NCCs. These findings are consistent with previously published expression patterns of *rpl5* in *Xenopus laevis* (Scholnick et al., 1997; Wischnewski et al., 2000; Session et al., 2016). Furthermore, *rpl5* expression is located in tissues which are mainly affected in patients suffering from ribosomopathies (Narla and Ebert, 2010; Farley-Barnes et al., 2019).

Here, we showed that the knockdown of Rpl5 results in a severe eye and brain phenotype. As shown in Figure 3E, the evagination of the eye vesicle is hindered upon Rpl5 depletion. This early disruption of the eye development most likely contributes later to a disturbed eye cup invagination and therefore contributes to a malformed eye. We found a disturbed retinal lamination. Interestingly, all cell types were present. Previous studies revealed that cell adhesion defects appearing as increased intercellular spaces cause this phenotype (Seigfried et al., 2017). As shown in Figure 3, *pax6* expression is reduced in Rpl5 morphants, which might contribute to these developmental defects in the retina, since it has been shown that several cell adhesion molecules are direct targets of Pax6 (Rungger-Brändle et al., 2010). Furthermore, Rax, which we found to be reduced in Rpl5 morphants, might affect proper retinal lamination since 1) Rax has been shown to impact retinal lamination in mice embryos (Rodgers et al., 2018) and 2) Rax-dependent genes have been shown to be necessary for proper retina lamination in *Xenopus* (Pan et al., 2018). Marker genes for the lens were not affected upon Rpl5 depletion suggesting that a disturbed development of the lens placodes does not contribute to the disorganized retinal lamination.

Ribosomal proteins other than Rpl5 also affect proper eye as well as brain development as Pescadillo homologue 1 (Pes1) and Peter Pan (Ppan) lead to malformed eyes, including coloboma, as well as microcephaly in developing *Xenopus laevis* (Gessert et al., 2007; Bugner et al., 2011). Watkins-Chow and others have shown that Rps7-mutated mice develop uveal coloboma and microphthalmia (Watkins-Chow et al., 2013). Mutations in human ribosomal proteins are also linked to defects in eye development (Kuze et al., 2009). Brain defects have been found in patients carrying a mutated Rpl10 or Rps23, as they show an increased incidence in microcephaly, seizures, aphasia, ataxia, and intellectual disability (Brooks et al., 2014; Bourque et al., 2018). In summary, these findings highlight the importance of Rpl5 in proper brain and eye development.

In order to gain insight into the observed defects on a molecular basis, marker gene expression was investigated at stages 13 and 23. Reduced expression of eye-specific marker genes *rax* and *pax6* implicate defects in early eye field induction as well as in eye cell differentiation in Rpl5-deficient embryos. Moreover, expression of the brain-specific marker genes *pax6*, *otx2*, and *egr2* was reduced upon Rpl5 depletion. The pan-neural maker gene *sox3*, however, was not affected, indicating that neural induction is not disturbed in general. In earlier studies we showed, that the depletion of Pes1 and Ppan also lead to reduced *pax6*, *rax*, and *otx2* expression in *Xenopus* (Gessert et al., 2007; Bugner et al., 2011). In mice, Pax6 depletion results in absent eyes (Georgala et al., 2011). In human, mutations in the *PAX6* gene are linked to a variety of eye defects such as aniridia, corneal opacification or cataract, as well as autism spectrum disorder (Maekawa et al., 2009; Cvekl and Callaerts, 2017). Not only *PAX6*, but also *OTX2*, *RAX*, and *EGR2* have been associated with eye and brain defects in humans (Gonzalez-Rodriguez et al., 2010; Nakayama et al., 2015; Sevilla et al., 2015; Deml et al., 2016). Taken together, these data indicate that the disturbed expression of these marker genes, as a result of Rpl5 knockdown, contribute to the eye and brain phenotype occurring in *Xenopus laevis* and that the induction as well as cell differentiation is perturbed. It would be highly interesting to analyze these genes in patients with a mutated *RPL5* gene and patients suffering from other ribosomopathies.

Following the induction of NCCs during neural tube closure, these cells migrate towards the ventral part of the body and build the cranial cartilage as one derivative (Jacobson, 1991). The reduced expression of NCC-specific marker genes, *twist1* and *snai2* at stages 15 and 20, shows that the induction as well as the migration of NCCs is impaired upon Rpl5 knockdown. These defects become apparent in the destructed cranial cartilage observed in Rpl5-deficient embryos at stage 45. Griffin et al. demonstrated in *Xenopus* embryos that knockdown of Nol11, another factor for ribosomal biogenesis, results in a reduced expression of several marker genes for cranial NCCs such as *twist1* (Griffin et al., 2015). Additionally, we showed a reduced expression of *snai2* and *twist1* during Ppan knockdown (Bugner et al., 2011).

In human, mutations in ribosomal proteins, like Rpl5, found in DBA patients have also been linked to craniofacial defects (Lipton et al., 2006). Furthermore, our observations are in line with Rps7 knockdown mice, showing skeletal defects, resulting in reduced body length (Watkins-Chow et al., 2013) as well as Rps19 and Rps20-mutated mice with pigmentation and skin defects (McGowan et al., 2008). In *Xenopus* embryos, previous studies moreover showed that knockdown of the ribosomal factors Pes1, Ppan, as well as Nol11 results in malformed cranial cartilages (Gessert et al., 2007; Bugner et al., 2011; Griffin et al., 2015).

Earlier studies revealed that the knockdown of ribosomal biogenesis factors, such as Ppan, results in early phenotypes—occurring as reduced marker gene expression—which are independent of ribosomal biogenesis in *Xenopus* (Bugner et al., 2011). Hence, *Xenopus laevis* is a suitable model organism to study ribosomal biogenesis factors and proteins in a ribosomal independent way. Since we here also observed a very early phenotype, we investigated the molecular mechanism underlying Rpl5 loss of function during *Xenopus*.

In this study, Rpl5 depletion led to a decreased number of proliferative cells. Since c-Myc is a major regulator of proliferative processes and ribosomal biogenesis, we analyzed the molecule c-Myc and showed a decrease in *c-myc* expression in the cranial NCCs, a partial rescue of the eye phenotype was achieved by co-injecting *c-myc* RNA, and a more than additive effect was found between loss of Rpl5 function and loss of c-Myc function. In line, Bellmeyer and others showed that the depletion of c-Myc results in deformed cranial cartilages in *Xenopus* embryos (Bellmeyer et al., 2003). According to Liao et al., free Rpl5 and Rpl11 proteins are capable of reducing *c-myc* RNA levels by directly binding *c-myc* RNA and transporting it to RISC eventually degrading *c-myc* RNA. The authors showed in a culture cell line that the knockdown of Rpl5 induces *c-myc* expression, whereas *rpl5* overexpression leads to *c-myc* degradation (Liao et al., 2014). Our findings are not in line with these results suggesting a different mechanism. Possibly, the depletion of Rpl5 might induce ribosomal stress, which eventually leads to a reduced *c-myc* expression by, e.g., free Rpl11 (Challagundla et al., 2011). Furthermore, Rpl5 may fulfill a role independent of ribosome biogenesis and affect c-Myc in a free-ribosomal way. Other mechanisms might be involved. However, based on our results that 1) *c-myc* expression is reduced upon Rpl5 depletion, 2) simultaneous loss of Rpl5 function and loss of c-Myc function leads to a more than additive effect, and 3) the Rpl5 MO-induced eye phenotype is partially rescued upon *c-myc* co-injection, we hypothesize a common signaling pathway of these two molecules, with c-Myc being downstream of Rpl5.

Furthermore, Rpl5 depletion was found to increase the number of apoptotic cells. To further analyze the mechanism underlying increased apoptosis, we investigated Tp53, a molecule which has been found to be accumulated and activated during disturbed ribosomal biogenesis by different ribosomal proteins (Dai and Lu, 2004); and which can initiate apoptosis as a transcription factor for genes necessary for apoptosis and by transcriptional-independent mechanisms, e.g., by directly targeting mitochondria inducing permeabilization of the mitochondrial membrane, which eventually leads to apoptosis (Kiraz et al., 2016). As mentioned above, several studies have shown that craniofacial abnormalities, which occurred during ribosomopathies, were rescued by knocking out *tp53* (Jones et al., 2008; Zhao et al., 2014; Griffin et al., 2015; Calo et al., 2018). In our study, however, we were not able to rescue the anterior neural phenotype by co-injecting Tp53 MO suggesting that the observed phenotype is not solely due to Tp53 activation as a consequence of disturbed ribosome biogenesis, e.g., through other free ribosomal proteins. This observation would be in line with the well accepted idea that early *Xenopus* embryos can rely on maternal ribosomes and do not require *de novo* synthesis of ribosomes. We observed, however, *tp53* expression to be increased on RNA level upon Rpl5 knockdown. In addition, the more than additive effect between Rpl5 knockdown and Tp53 overexpression suggests a common signaling pathway between those two molecules. Furthermore, by co-injecting *hBCL2* RNA we were able to partially rescue the Rpl5 MO-induced eye phenotype. The proto-oncogene *BCL2* is a blocker of apoptosis and mutations in the *BCL2* gene lead to cancer (Reed, 2008). In line with our results, it has been shown that Tp53 directly or indirectly blocks antiapoptotic BCL2 contributing to further cell death (Hemann and Lowe, 2006). Hence, the possible Bcl2 inhibition by Tp53, which is induced by Rpl5 depletion, can be counteracted by co-injection of *hBCL2* RNA in rescue experiments. This suggests that increased apoptosis is to a great extent contributor to the here observed phenotypes in *Xenopus laevis*.

Based on our results that *tp53* expression is increased in the anterior tissue during early embryonic development and *hBCL2* co-injection rescued the Rpl5 MO-induced phenotype, it is conceivable that continuous cell death induced by Tp53 pathway activation may lead to the different morphogenesis phenotypes.

Although it is widely accepted that early *Xenopus* embryos can rely on maternal ribosomes and thus do not require ribosome biogenesis during early stages of development, another explanation of the observed phenotypes might also be possible. In line with the different morphogenesis phenotypes are also the so-called ribosome concentration theory and the “specialized” ribosomes hypothesis. In the first case one could assume that maternal ribosomes are not equally distributed to all cells of the embryo resulting in cells that require *de novo* ribosome synthesis earlier than others. According to this theory, the translation of specific mRNAs may also depend on ribosome concentration and therefore again translational landscape might be altered (Lodish, 1974; Mills and Green, 2017; Cheng et al., 2019; Farley-Barnes et al., 2019). “Specialized” ribosomes are one hypothesis, in which already naturally existing heterogeneous ribosomes further acquire diverse abilities in distinct tissues due to changes in ribosomal assembly and composition (Mills and Green, 2017; Genuth and Barna, 2018; Ferretti and Karbstein, 2019; Norris et al., 2021). Both theories might contribute to the different morphogenesis phenotypes but are not yet investigated in *Xenopus* embryos.

Taken together, our findings indicate a role of Rpl5 for early *Xenopus* neural development and should foster additional experiments to examine the potential underlying mechanisms.

## Data Availability

The original contributions presented in the study are included in the article/Supplementary Material, further inquiries can be directed to the corresponding author.

## Supplementary Material

Supplementary Figure S1 Rpl5 synteny analysis and protein comparison between different species. **(A)** Synteny analysis of *rpl5* in *Homo sapiens*, *Mus musculus*, *Gallus gallus*, *Xenopus laevis L*, *Xenopus laevis S*, *Xenopus tropicalis*, and *Danio rerio*. Comparing the genomic region next to *rpl5* showed a conservation across the different species. **(B)** Protein alignment of Rpl5 of the different species *Homo sapiens*, *Mus musculus*, *Gallus gallus*, *Xenopus laevis L*, *Xenopus laevis S*, *Xenopus tropicalis*, *Danio rerio*. Protein homology is given in % compared to *Homo sapiens*. Abbreviations: aa, amino acid; Rpl5, ribosomal protein L5.

Supplementary Figure S2 Spatial and temporal expression of *rpl5* in *Xenopus laevis* (extended). **(A)** *rpl5* expression was analyzed in wildtype stage 23 embryos *via* WMISH. Lateral views and sections are shown. *rpl5* transcripts are enriched in migrating NCCs (white arrow), the developing eye (red arrow), and somites (orange arrowhead). White dotted line represents level of transversal sections in **(a1,2)**, where *rpl5* expression was found in the neural tube and somites (indicated by black dotted lines). *actc1* was used as marker gene for somites. Section orientation is dorsal (upper part) to ventral (lower part). **(B)** Ventral view of the embryos is given. At stage 28, embryos show *rpl5* expression in the ventral blood islands (blue arrowhead), where *gata2* and *hba3* are expressed as well. Abbreviations: nc, notochord; nt, neural tube; s, somites.

Supplementary Figure S3 Morpholino oligonucleotide binding affinity assay. **(A)** Binding sites (bs) of Rpl5 MO and the *Δ5′UTR rpl5* construct on *Xenopus rpl5*. Blue letters indicate the start codon. Grey letters indicate differences between Rpl5 MO binding site and the Δ5′UTR rpl5 construct. **(B)** Binding specificity test of Rpl5 MO. Co-injection of 1 ng *rpl5 MO bs-GFP* together with 10 ng Control MO led to GFP expression. 1 ng *rpl5 MO bs-GFP* injected with 10 ng Rpl5 MO efficiently blocked GFP translation. Co-injection of *Δ5′UTR rpl5 MO bs-GFP* along with 10 ng Rpl5 MO, however, led to GFP expression. Abbreviations: bs, binding site; CoMO, Control morpholino oligonucleotide; GFP, green fluorescent protein; n, number of independent experiments; N, number of injected embryos and analyzed; Rpl5 MO, ribosomal protein L5 morpholino oligonucleotide; UTR, untranslated region.

Supplementary Figure S4 Retinal lamination upon Rpl5 knock down in detail. Analysis of retinal lamination specific marker genes, *rho*, *prox1*, *vsx1*, *pax6*, and *pou4f1* in Control MO and Rpl5 MO-injected embryos at stage 43. The entire eye of the uninjected and injected side are shown as well as a detailed view (indicated by black boxes) of the respective cell layer. Control MO injection did not alter the retinal lamination. Rpl5 depletion led to mild and severe phenotypes with disturbed retinal layer and malformed eyes. Number below the columns indicate the number of embryos showing the depicted phenotype per number of embryos analyzed. Abbreviations: CoMO, Control morpholino oligonucleotide; inj., injected; Rpl5 MO, ribosomal protein L5 morpholino oligonucleotide; uninj., uninjected.

Supplementary Figure S5 Rpl5 depletion affects erythropoiesis. **(A)** 15 ng Control MO or Rpl5 MO were injected into one ventral blastomere of eight-cell stage embryos to target blood islands. Ventral view of embryos is given. Embryos were fixed at stage 28 and *gata2* expression was analyzed *via* WMISH. **(B)** *gata2* expression was not affected by either Rpl5 MO or Control MO injection. **(C)** Embryos were injected with 15 ng Control MO or Rpl5 MO into one ventral blastomere at eight-cell stage. Morphants were fixed at stage 28 and *hba3* expression was analyzed by WMISH. Ventral view of embryos is shown. Embryos injected with Rpl5 MO show a reduced *hba3* expression on the injected side (indicated by black arrowhead), whereas Control MO injection did not reduce *hba3* expression. **(D)** Statistical analysis of data shown in **(C)**. Abbreviations: CoMO, Control morpholino oligonucleotide; inj., injected; n, number of independent experiments; N, number of injected embryos and analyzed; n.s., non-significant; Rpl5 MO, ribosomal protein L5 morpholino oligonucleotide; uninj., uninjected. Error bars indicate standard error of the means; **p* ≤ 0.05.

Supplementary Figure S6 Combined injection of Rpl5 MO and c-Myc MO did not alter proliferation and simultaneous injection of Rpl5 MO and Tp53 MO did not rescue the Rpl5 MO-induced phenotype. **(A)** Embryos were injected with 5 ng Rpl5 MO, 5 ng c-Myc MO or 5 ng Rpl5 MO together with 5 ng c-Myc MO into one animal-dorsal blastomere at eight-cell stage. Embryos were fixed at stage 23 and proliferative cells were stained with a pH3 antibody. Anterior views are shown. Number of proliferative cells was counted (black boxes indicate area where pH3 positive cells were counted) and pH3 positive cells on the injected sides were compared between the three different conditions. **(B)** The number of proliferative cells did not alter between Control MO, Rpl5 MO, and Rpl5 MO together with c-Myc MO injection. **(C)** Control MO (17.5 ng or 20 ng), Rpl5 MO (15 ng), and Rpl5 MO (15 ng) + Tp53 MO (2.5 ng or 5 ng) were injected into eight-cell stage embryos to directly target anterior neural tissue. Embryos were fixed at stage 43 and analyzed regarding smaller eyes or heads (white arrows). **(D)** Statistical analysis showed that Rpl5 MO alone as well as in combination with Tp53 MO resulted in a severe eye and head phenotype. The here tested Tp53 MO amount did not rescue the Rpl5 MO-induced phenotype. **(E)** 17.5 ng Control MO, 15 ng Rpl5 MO, or 15 ng Rpl5 MO together with 2.5 ng Tp53 MO were injected at eight-cell stage. Embryos were fixed at stage 43 and subsequently photographed. Area of the eye (red dotted line) was measured and the injected side was compared to the uninjected side. **(F)** Rpl5 MO injection alone and in combination with Tp53 MO resulted in significantly smaller eyes on the injected side. Control MO injection did not reduce eye size. **(G)** 20 ng Control MO, 15 ng Rpl5 MO, or 15 ng Rpl5 MO together with 5 ng Tp53 MO were injected at eight-cell stage. Embryos were fixed at stage 43 and subsequently photographed. Area of the eye (red dotted line) was measured and the injected side compared to the uninjected side. **(H)** Control MO injection did not reduce the eye area. Rpl5 MO injection alone as well as together with Tp53 MO results in smaller eye areas. Tp53 depletion did not rescue the Rpl5 MO-induced phenotype in the here tested conditions. Abbreviations: c-Myc MO, Myc proto-oncogene morpholino oligonucleotide; CoMO, Control morpholino oligonucleotide; inj., injected; n, number of independent experiments; N, number of injected embryos and analyzed; n.s., non-significant; pH3, phospho histone 3; Rpl5 MO, ribosomal protein L5 morpholino oligonucleotide; Tp53 MO, Tumor protein p53 morpholino oligonucleotide; uninj., uninjected. Error bars indicate standard error of the means; **p* ≤ 0.05; *****p* ≤ 0.0001.

## Abbreviations

c-myc, MYC proto-oncogene; DBA, Diamond Blackfan anemia; DEPC, diethyl pyro carbonate; hBCL2, human B cell lymphoma 2; MDM2, mouse double minute 2 homolog; MO, morpholino oligonucleotide; NCC, neural crest cell; Pes1, Pescadillo homologue 1; pH3, phospho histone 3; Ppan, Peter pan; RISC, RNA-induced silencing complex; RNP, ribonucleoprotein; RPE, retinal pigmented epithelium; Rpl5, ribosomal protein L5; Rpl11, ribosomal protein L11; RT, reverse transcriptase; Tp53, tumor protein p53; TUNEL, terminal deoxynucleotidyl transferase dUTP-biotin nick end labeling; WMISH, whole mount *in situ* hybridization.
