# Supplementary figures and images for: The Ribosomal Protein L5 Functions During Xenopus Anterior Development Through Apoptotic Pathways
Source: Front Cell Dev Biol. 2022 Feb 22;10:777121. doi: 10.3389/fcell.2022.777121 (PMC8905602; doi:10.3389/fcell.2022.777121)

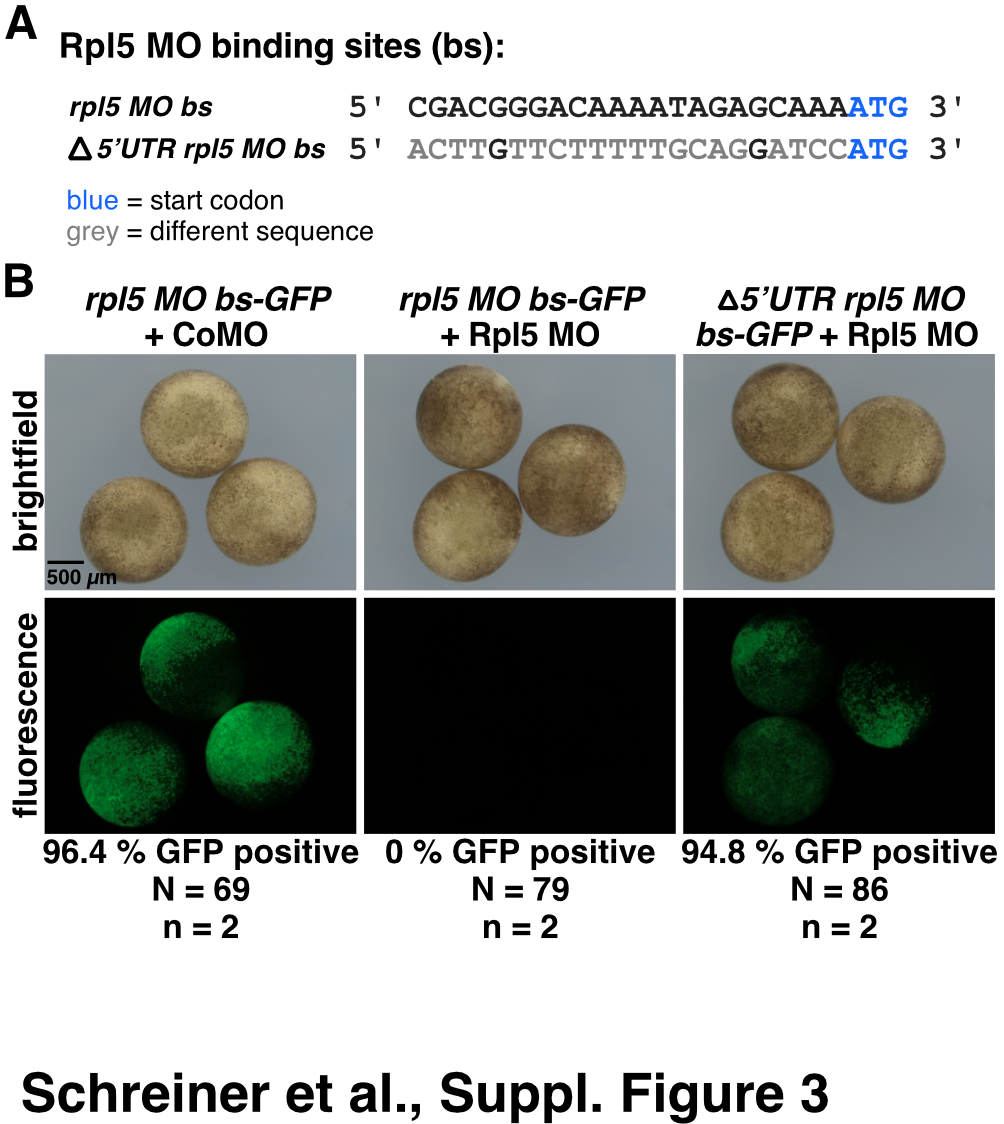

Supplement: Supplementary file 1 [file Image3.TIFF]

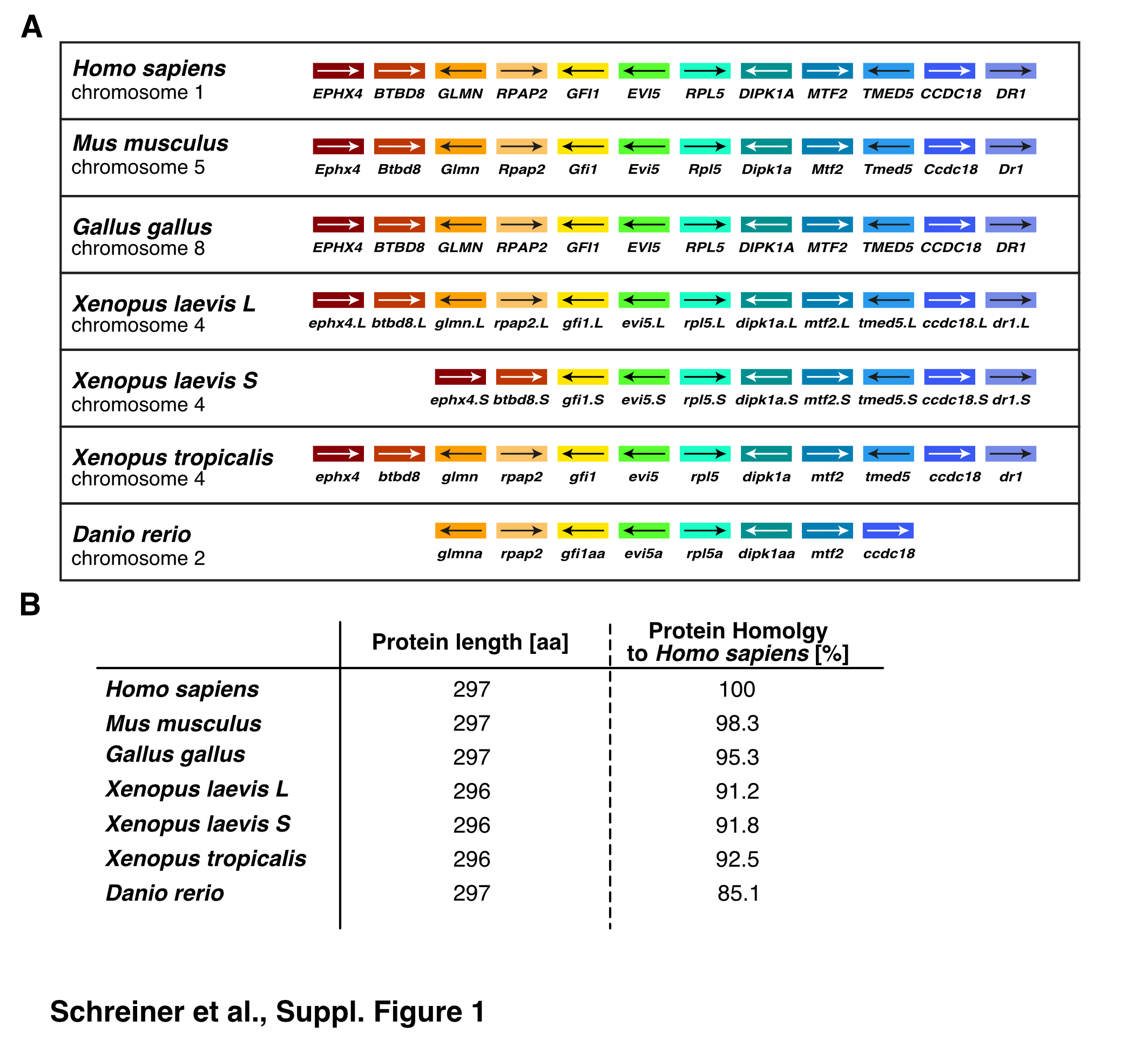

Supplement: Supplementary file 2 [file Image1.TIFF]

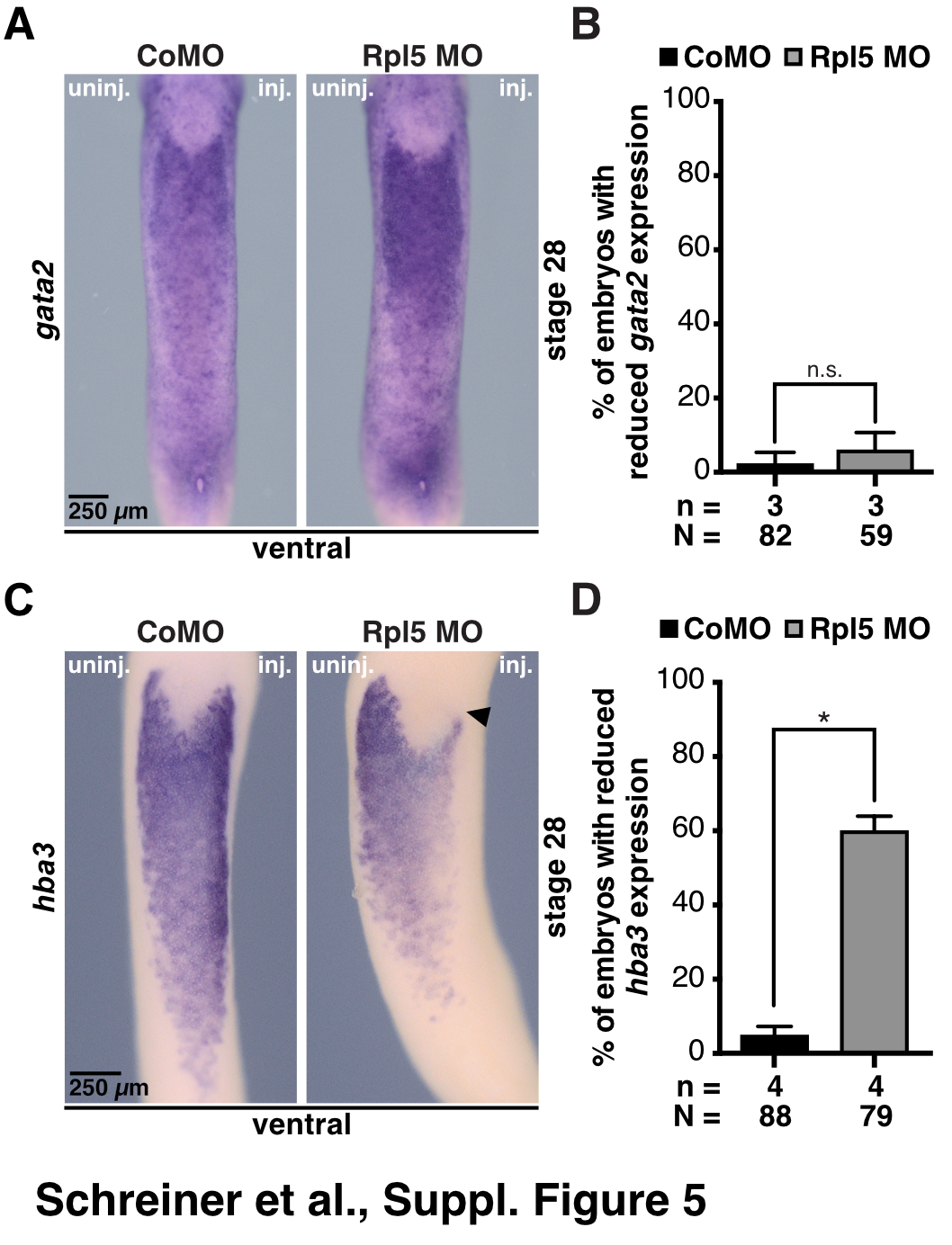

Supplement: Supplementary file 3 [file Image5.TIFF]

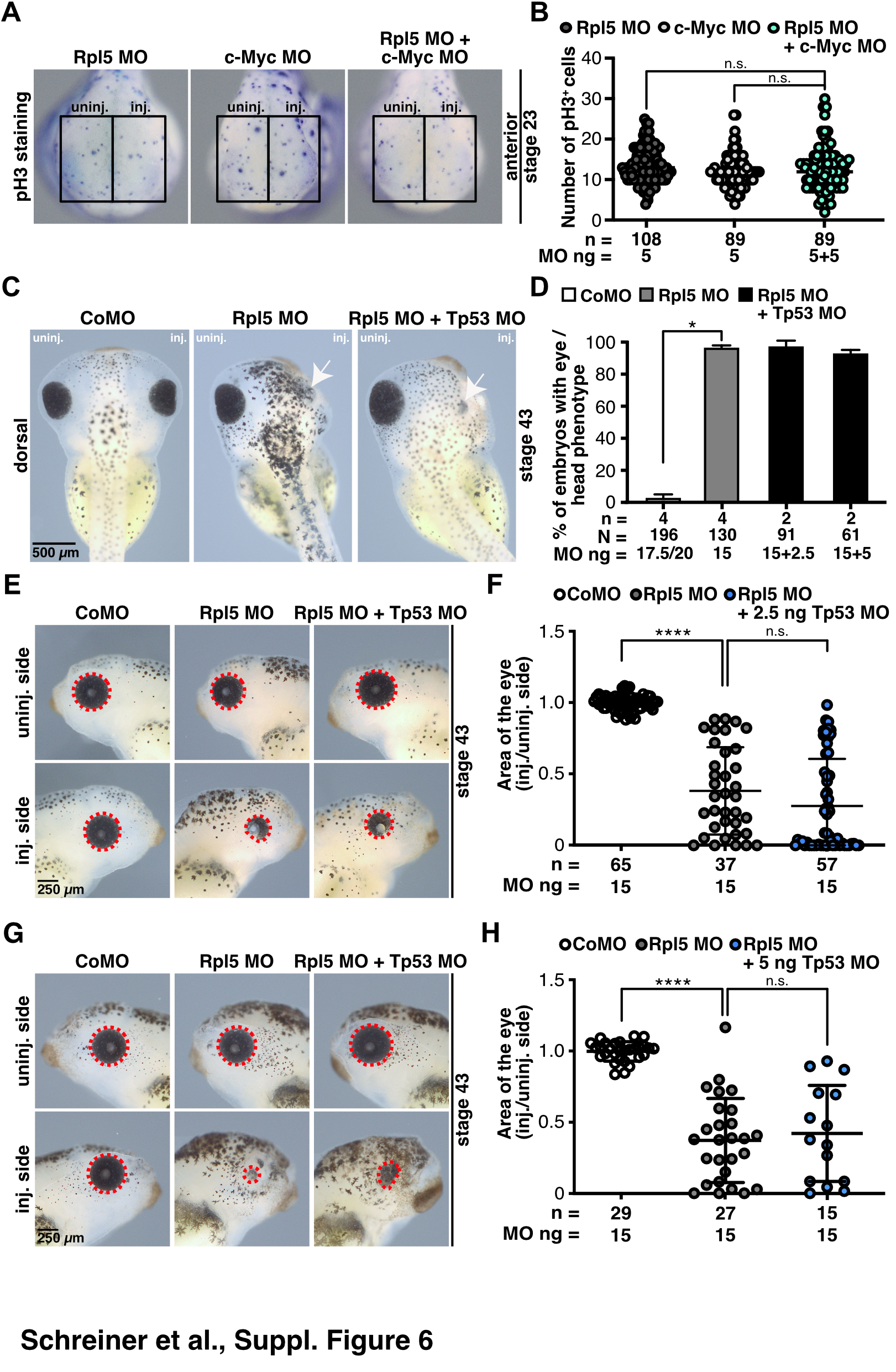

Supplement: Supplementary file 4 [file Image6.TIFF]

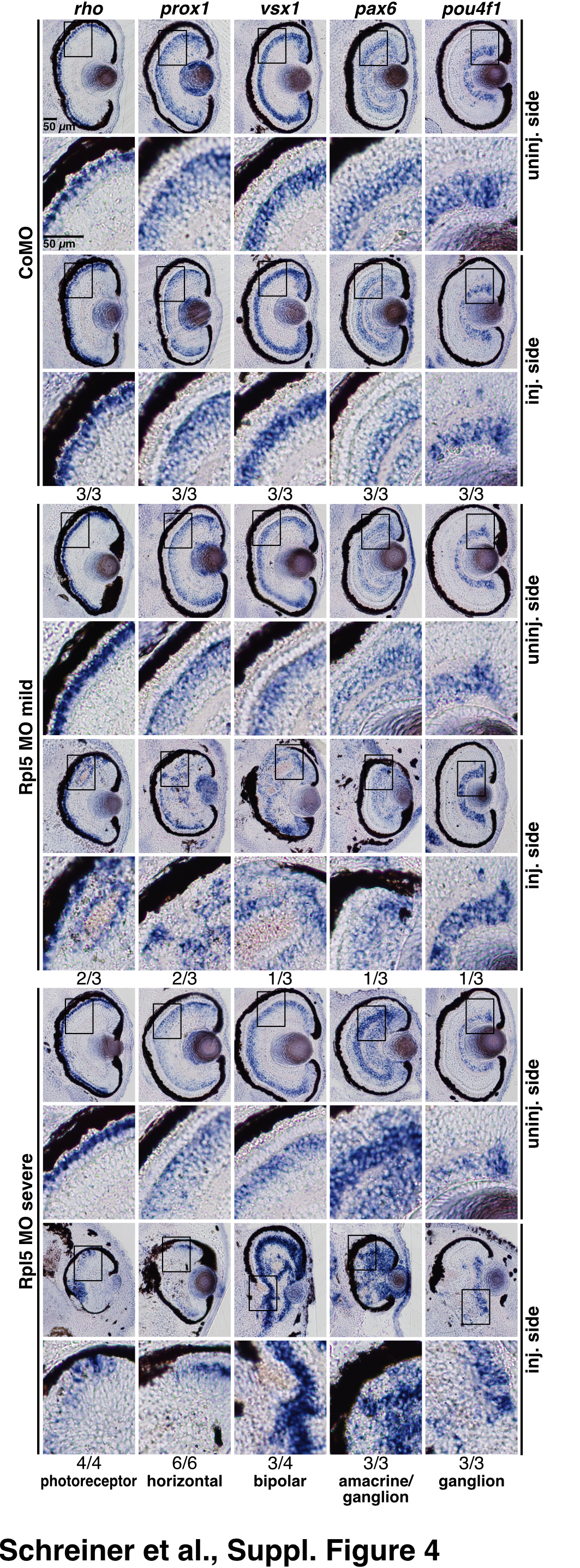

Supplement: Supplementary file 5 [file Image4.TIFF]
